# Advancements in Ocular Neuro-Prosthetics: Bridging Neuroscience and Information and Communication Technology for Vision Restoration

**DOI:** 10.3390/biology14020134

**Published:** 2025-01-28

**Authors:** Daniele Giansanti

**Affiliations:** Centro TISP, ISS Via Regina Elena 299, 00161 Rome, Italy; daniele.giansanti@iss.it

**Keywords:** ocular neuro-prosthesis, ocular bionic prosthesis, bionic eye, artificial retina, artificial vision, artificial intelligence

## Abstract

Background: Neuroprosthetics for vision restoration have advanced significantly, incorporating technologies like retinal implants, cortical implants, and non-invasive stimulation methods. These advancements hold the potential to tackle major challenges in visual prosthetics, such as enhancing functionality, improving biocompatibility, and enabling real-time object recognition. Aim: The aim of this review overview is to provide a comprehensive analysis of the latest advancements in ocular neuroprostheses. Methods: A narrative review was conducted, focusing on the latest developments in visual neuroprosthetics. Comprehensive searches were carried out on Google Scholar, PubMed, and Scopus using specific keywords. A specific narrative checklist was applied, alongside a tailored quality assessment methodology, to evaluate the quality of the studies included. A total of sixteen relevant studies from the past three years were included in the review. Results and discussion: The integration of artificial retinas, cortical implants, high technology-enabled prosthetics, gene therapies, nanotechnology, and bioprinting has shown significant promise in enhancing the quality and functionality of vision restoration systems, offering potential to address complex visual impairments and improve independence and mobility for individuals with blindness. These innovations appear to have the potential to transform healthcare systems in the future by enabling more efficient and personalized therapies and prosthetic devices. However, challenges such as energy efficiency, scalability, and the neural dynamics of vision restoration persist, requiring continued interdisciplinary collaboration to refine these technologies, overcome ethical and regulatory hurdles, and ensure their effectiveness in real-world applications. Conclusions: While visual neuroprosthetics have made remarkable progress, addressing challenges related to energy consumption and regulatory and ethical concerns will be crucial for ensuring that neuroprosthetic devices can effectively meet the needs of individuals with visual impairments.

## Simple Summary

Neuro-prosthetics for vision restoration have advanced with technologies like retinal and cortical implants and non-invasive stimulation methods, aiming to enhance functionality and real-time object recognition. A narrative review of 16 recent studies highlights significant progress and the contributions of emerging approaches such as nanotechnology, bioprinting, and AI. Despite these advancements, challenges remain, including energy efficiency, scalability, biocompatibility, ethical and regulatory concerns, and understanding neural dynamics. Addressing these issues requires ongoing interdisciplinary collaboration to refine technologies and ensure their effectiveness in real-world applications. While progress has been remarkable, overcoming these barriers is crucial to meet the needs of individuals with visual impairments and fully realize the potential of neuroprosthetic devices.

## 1. Introduction

### 1.1. Background

Blindness is a condition characterized by the complete or partial loss of vision, significantly impacting the quality of life and independence of those affected. It can be congenital, stemming from genetic disorders, or acquired due to conditions such as retinitis pigmentosa, glaucoma, or macular degeneration. According to the World Health Organization (WHO) [1], approximately 43 million people worldwide live with blindness, while over 1 billion experience visual impairments that could be prevented or treated.

Blindness not only restricts visual perception but also affects psychological, social, and economic aspects of life. Despite these challenges, blind individuals often exhibit remarkable adaptability, honing advanced skills in other senses, such as touch and hearing.

The WHO also emphasizes the role of assistive technologies in improving the quality of life for people living with blindness and visual impairments [2]. The WHO highlights that assistive devices improve independence and mobility while also supporting education, employment, and social inclusion for individuals with vision loss. Access to these technologies is considered a fundamental aspect of universal health coverage and human rights, as highlighted in their reports on assistive technology and its transformative impact for blindness and visual impairment. The National Council on Aging (NCOA) [3], a resource hub and U.S.-based nonprofit organization that works to improve the lives of older adults, providing resources, advocacy, and programs to enhance health, independence, and financial security, highlights the role of assistive technologies and vision rehabilitation services in helping people maintain independence and improve their quality of life. According to NCOA, specific ATs and training include:

Assistive Devices: Tools like screen readers, magnification software, Braille displays, and specialized apps that convert text to speech or describe the surroundings audibly.

Orientation and Mobility Training: Teaches individuals to navigate their environment safely and confidently using tools like white canes, GPS systems, or guide dogs.

Daily Living Skills Training: Helps individuals learn or relearn tasks such as cooking, personal care, and managing medications safely.

Vision Rehabilitation Services: Includes low-vision exams, home modifications, and counseling to address mental health challenges associated with vision loss.

These resources aim to enhance independence and reduce the social isolation often experienced by individuals with vision impairments.

However assistive technologies (ATs) do not aim to restore lost vision but rather supplement it using alternative sensory channels, most commonly hearing. These technologies fall under the category of augmentative and alternative communication (AAC), which seeks to improve the lives of people with disabilities by utilizing available sensory faculties such as hearing, touch, or even proprioception (the sense of body position) [4]. For example, screen readers convert text into speech, while apps using augmented reality can provide auditory feedback about the surrounding environment. This approach does not restore sight but instead supports independence and functional navigation by tapping into other senses, thus improving the user’s interaction with their environment. Examples include technologies like smart canes, which can give haptic or auditory feedback about obstacles.

On the other hand, ocular prostheses, such as artificial eyes or implants, have a long history rooted more in aesthetic restoration rather than functional vision restoration. These prostheses were initially developed to conceal the disability caused by eye loss, and their primary goal was cosmetic—to restore the outward appearance of the eye and help individuals reintegrate into social settings with fewer visible signs of disability.

Cosmetic ocular prostheses [5,6] are designed primarily to improve appearance after the loss of an eye, but they do not restore vision. Their primary function is aesthetic enhancement, offering individuals a means of restoring a more natural appearance and maintaining social confidence. These prostheses come in various forms [7]:

Acrylic prostheses: They are custom-made from durable acrylic material to replicate the appearance of a healthy eye, designed for a comfortable fit while matching the color and shape of the remaining eye to provide a realistic look; while they do not restore vision, they significantly enhance a person’s outward appearance.

Glass Eye: This was a traditional version of the ocular prosthesis, which is less commonly used today. While it serves a similar cosmetic purpose, glass prostheses are more fragile and less customizable than acrylic versions. The shift to acrylics has occurred due to better durability and ease of customization.

Prostheses with Integrated Movement: These prostheses are more advanced and connect to the residual ocular muscles [8]. By using these muscles, they mimic the movement of a healthy eye, allowing for more natural eye movement and providing a more lifelike appearance. Although they still do not restore vision, these prostheses can help individuals feel more natural in their daily interactions.

While these prosthetics do not restore visual function, they significantly enhance the psychological and social well-being of individuals by helping them reintegrate into social environments with greater comfort and confidence.

Advancements in ocular prosthetics:

The field of visual prosthetics has seen remarkable progress, evolving from purely cosmetic solutions into systems aimed at restoring functional vision. Initially, prosthetic devices were designed primarily to address aesthetic concerns for individuals who had lost an eye, helping to mask physical impairments. However, with the advent of modern technology, the focus has shifted toward devices that actively engage with the nervous system to provide some degree of visual perception.

These new prostheses aim to stimulate specific parts of the visual system, helping individuals with vision loss caused by damage to the retina, optic nerve, or visual cortex. Unlike their predecessors, these devices are not only concerned with appearance but also with function, striving to enhance spatial awareness and perception. By bypassing damaged areas of the visual pathway, these systems introduce a new era of functional integration with the nervous system.

In recent years, advancements in biotechnology, neuroscience, and information and communication technologies (ICTs) have given rise to a specialized subfield: visual neuroprosthetics. This domain merges the precision of electronic engineering with the adaptability of the human brain, creating devices that interact seamlessly with the visual and neurological systems. By leveraging breakthroughs in areas such as microelectronics, neuroplasticity, and bioengineering, visual neuroprosthetics are extending the possibilities for restoring vision.

These technologies represent an interdisciplinary effort to address the complexities of vision loss. They range from systems that stimulate residual sensory structures, such as retinal ganglion cells, to advanced interfaces that directly connect with the visual cortex, enabling some perception even when intermediate pathways are nonfunctional. This progression is best understood through a classification into five main categories, which reflect the diverse approaches to visual restoration: retinal prostheses, cortical prostheses, hybrid systems, regenerative prostheses, and non-invasive stimulation techniques. Together, these categories (Table 1) illustrate the potential of visual neuroprosthetics to transform lives by reconnecting individuals to their environment in ways previously thought impossible.

Retinal Prostheses:

Retinal prostheses [9,10] are specifically designed for individuals with degenerative retinal diseases, such as retinitis pigmentosa and macular degeneration, where the light-sensitive cells (photoreceptors) of the retina are damaged. These prostheses work by stimulating the remaining retinal cells, like the ganglion cells, using electrical impulses. For example, the Argus II system sends electrical signals to the retinal ganglion cells, enabling the perception of light and basic shapes. Another example is the PRIMA System, which uses a microchip implanted under the retina to convert light into electrical impulses, stimulating the retina and partially restoring sight. While these devices do not provide full restoration, they help users detect visual cues, improving spatial awareness and mobility.

Cortical Prostheses:

For individuals with total blindness due to damage to the optic nerve or visual cortex, cortical prostheses are used [11,12]. These devices bypass the eye and directly stimulate the visual cortex in the brain, which is responsible for processing visual information. The Orion system is an example of a cortical prosthesis that stimulates the visual cortex directly, allowing individuals to perceive basic visual stimuli, like light or shapes, even if their optic nerve is completely damaged. While these devices do not provide detailed or high-resolution vision, they can enhance spatial awareness and help users navigate the world.

Hybrid or Experimental Prostheses:

The integration of multiple technologies has led to the development of hybrid prostheses [13,14], which combine retinal implants with external devices to improve visual recognition and perception. For instance, smart glasses can work alongside retinal implants to enhance face recognition and object identification, helping users interact more effectively with their environment. Additionally, optogenetic prostheses utilize light-sensitive genes to make retinal cells responsive to light, offering a potential solution for individuals with retinal degeneration by bypassing the damaged photoreceptors.

Biological or Regenerative Prostheses:

Emerging technologies also focus on biological or regenerative prostheses [15], which aim to restore vision through the regeneration or replacement of damaged tissues. Stem cell therapy is being explored to regenerate retinal cells, while bioengineered tissues may be used to create artificial retinas or corneas. The use of nanotechnology is also being investigated, as it could enable interaction with neurons at a microscopic level, potentially aiding in the regeneration of damaged eye tissues and enhancing vision at the cellular level.

Non-Invasive Electrical Stimulation:

In addition to invasive prostheses, non-invasive electrical stimulation is being studied [16,17] as a way to enhance residual vision in people with low vision. These systems typically use electrical impulses to stimulate the optic nerve or other parts of the visual pathway, improving the perception of light, shapes, or contrast. These devices are less invasive than implantable systems and offer a promising option for individuals who still retain some degree of functional vision but need assistance to enhance their visual processing.

By integrating advancements from human sensory research to brain–computer interface technology, neuroprosthetics provide a unified framework for addressing vision loss at different levels of the visual system. These categories collectively demonstrate how cutting-edge science is bridging the gap between biological systems and digital innovation, offering unprecedented hope for individuals with visual impairments.

The field of functional visual neuroprostheses is evolving rapidly, combining various techniques and technologies to improve the lives of individuals with vision loss. Although full restoration of sight remains a challenging goal, these devices are already helping individuals regain a degree of visual perception, enhancing their ability to navigate the world and interact with others. Through ongoing research and development, there is growing optimism that future advancements may lead to even more research in visual impairments [18].

**Table 1:** Sketch of type of ocular neuroprosthesis (*).

Type of Neuroprosthesis	Explanation of Why It is a Neuroprosthesis	Description
Retinal Prostheses	These devices are designed to stimulate the retina, which is part of the nervous system, to restore vision. They directly interface with the retinal ganglion cells, bypassing damaged photoreceptors.	Retinal prostheses like the Argus II and PRIMA System restore basic vision for individuals with retinal degenerative diseases (e.g., macular degeneration, retinitis pigmentosa). They provide electrical impulses to stimulate remaining retinal cells, allowing users to perceive light and basic shapes, which improves spatial awareness and mobility.
Cortical Prostheses	These prostheses bypass the eye and optic nerve, stimulating the visual cortex of the brain directly, which is part of the central nervous system responsible for processing visual information.	Cortical prostheses like the Orion system stimulate the visual cortex in individuals who have complete blindness due to optic nerve or cortical damage. These devices allow the perception of basic visual stimuli (light, shapes) by bypassing damaged eye structures, though they do not restore detailed vision. They enhance spatial awareness and navigation for users.
Hybrid or Experimental Prostheses	These combine retinal implants with external technologies to enhance visual processing. They interact with the nervous system to improve overall vision restoration.	Hybrid prostheses integrate retinal implants with external devices (e.g., smart glasses or optogenetics) to enhance vision. They may use technologies like light-sensitive genes or external visual aids to help users recognize faces or objects more effectively. These devices aim to enhance recognition and perception by bridging the gap between biological and external systems.
Biological or Regenerative Prostheses	These systems focus on regenerating or replacing damaged retinal or eye tissues, interacting with the nervous system and helping restore vision at the cellular level through stem cells or bioengineered tissues.	Regenerative prostheses use stem cell therapy or nanotechnology to regenerate damaged retinal cells or create bioengineered tissues such as artificial retinas or corneas. These technologies aim to repair the visual pathway, potentially restoring vision by regenerating the biological components of the eye or restoring lost tissue functionality.
Non-Invasive Electrical Stimulation	These techniques stimulate the optic nerve or visual pathway without invasive procedures, enhancing visual processing and improving residual vision through external stimulation.	Non-invasive electrical stimulation devices apply electrical impulses to stimulate the optic nerve or parts of the visual system, improving perception of light, shapes, or contrast. These devices are designed for individuals with low vision who still retain some functional sight but need assistance to enhance their visual capabilities.

* Each of these neuroprosthetic technologies aims to restore vision by directly or indirectly stimulating components of the visual pathway within the nervous system, offering hope for individuals with severe visual impairments. They represent the intersection of cutting-edge neuroscience and prosthetics, advancing our understanding of how technology can restore sensory functions previously thought impossible.

### 1.2. Purpose of the Umbrella Review

An overview of reviews in the field of visual neuroprosthetics can serve as a valuable resource, synthesizing current research trends, identifying gaps, and guiding future investigations. To provide a comprehensive understanding, such a review should address critical questions that explore the technological, biological, and clinical dimensions of this rapidly evolving field.

To guide an overview of reviews in the field of visual neuroprosthetics, three central questions can frame the inquiry effectively:

- What are the current technologies and their mechanisms in visual neuroprosthetics?
  This question seeks to outline the landscape of existing devices, from retinal and cortical prostheses to hybrid systems, and explain how they work. It includes exploring the mechanisms of electrical stimulation, biological regeneration, and optogenetics, as well as how these technologies address specific causes of vision loss.
- What clinical outcomes and challenges are associated with visual neuroprosthetics?
  This addresses the effectiveness of these devices in restoring vision and improving quality of life. It includes questions of functional success, patient satisfaction, and the technical or biological barriers that hinder widespread adoption, such as device reliability, neural adaptation, and integration with the nervous system.
- What are the emerging trends and future directions in this field?
  This question highlights cutting-edge research, including experimental approaches like nanotechnology and stem cell therapies. It also explores the role of interdisciplinary collaboration, ethical considerations, and the potential for new technologies to reshape the field.

By addressing these questions, an overview can not only synthesize the current state of the art but also offer a roadmap for future innovations and solutions in visual neuroprosthetics.

The general aim of this review is to synthesize existing knowledge on visual neuroprosthetics, with a particular focus on their integration within the health domain and their potential to address vision loss. This work seeks to provide a holistic perspective on the state of the field, covering its technological, clinical, and research dimensions to better understand its current applications and future opportunities.

The specific sub-aims are:

1. Trends: Examine the evolution and trends in the research in this field.
2. Categorization: Create a comprehensive classification of studies, emphasizing the emerging themes and categorization
3. Opportunities and Challenges: Evaluate the opportunities and challenges associated with visual neuroprosthetics, including their ethical, regulatory, and technical considerations, as well as their potential for widespread adoption and integration into healthcare systems.

This overview aims to serve as a valuable resource for researchers, clinicians, and policymakers, consolidating the current state of knowledge, identifying critical gaps in the literature, and establishing priorities for future research and development in visual neuroprosthetics.

## 2. Methods

An overview of reviews (narrative review of reviews) was conducted, focusing on the field. A standardized checklist for narrative reviews, the ANDJ Narrative Checklist, available online, was used [19]. The search was based on targeted searches on Google Scholar, PubMed, and Scopus. The search was based on targeted searches on Google Scholar, PubMed, and Scopus based on both full text and [Title/abstract].

The identified search key was the following:

(((“ocular”[All Fields] OR “oculars”[All Fields]) AND “neuroprosthesis”[Title/Abstract]) OR “retinal implants”[Title/Abstract] OR “bionic eyes”[Title/Abstract] OR ((“vision s”[All Fields] OR “vision, ocular”[MeSH Terms] OR (“vision”[All Fields] AND “ocular”[All Fields]) OR “ocular vision”[All Fields] OR “vision”[All Fields] OR “visions”[All Fields] OR “visioning”[All Fields]) AND “prosthetics”[Title/Abstract]) OR “artificial retina”[Title/Abstract] OR “optical neural implants”[Title/Abstract])

We also used a consolidated approach for the assessment criteria in the inclusion.

The assessment criteria for study inclusion in the narrative review to ensure rigor and quality were based on the following assessment.

Clarity of Rationale (N1): Evaluates whether the study clearly states the research problem, its significance, and why the investigation is necessary.

Design Appropriateness (N2): Assesses if the study design is suitable for answering the research question, including factors like methodology, sample size, and data collection methods.

Methodological Clarity (N3): Refers to how clearly the study’s methods are described and whether they are replicable, with transparency in data collection, analysis, and interpretation procedures.

Result Presentation (N4): Focuses on how well the study presents its findings, ensuring the results are clearly organized, accurately reported, and properly interpreted.

Justification of Conclusions (N5): Examines whether the conclusions are logically supported by the results, including discussions on the implications, limitations, and suggestions for future research.

Disclosure of Conflicts of Interest (N6): Checks if the authors have declared any conflicts of interest to assess the study’s impartiality and credibility.

Studies were rated on a scale of 1 to 5 for N1–N5, with a binary (Yes/No) assessment for N6. Only studies with “Yes” for N6 and a score greater than 3 for N1–N5 were selected.

Additionally, more recent reviews in the last three years were prioritized to ensure the inclusion of the latest advancements in the field.

The prioritization of more recent reviews (from the last three years) was carefully considered to ensure that the overview remains current and reflects the latest advancements in the field. Visual prosthetics are a rapidly evolving area, with technologies such as AI-driven systems, non-invasive stimulation techniques, and innovative materials being integrated into new prosthetic designs. These developments have the potential to significantly improve device functionality and user experience, addressing challenges such as real-time object recognition, user comfort, and biocompatibility. Recent publications are more likely to address these innovations, offering insights into cutting-edge methodologies and emerging trends. Additionally, prioritizing recent studies ensures that the review captures the most up-to-date data, addressing any gaps left by earlier reviews and providing a comprehensive understanding of current challenges and opportunities in visual prosthetics. This strategic focus on newer studies enhances the relevance and accuracy of the review’s findings.

**Figure 1:**
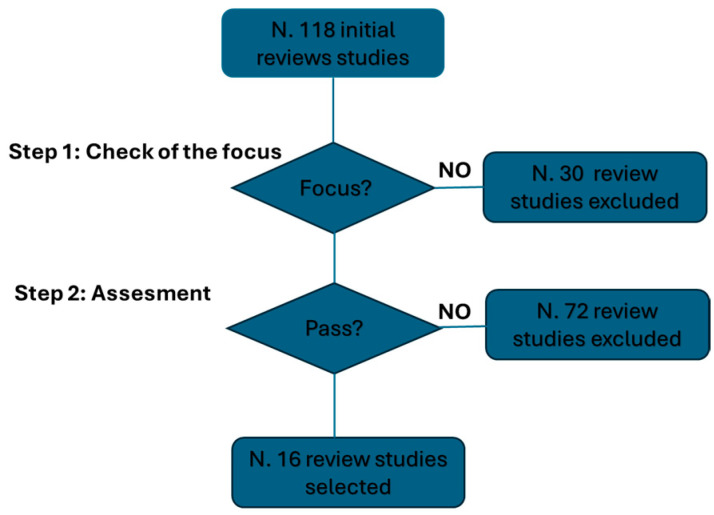
Sketch of the process selection of the review studies.

Figure 1 shows the process of selecting review studies based on the focus and the selection procedure, which also prioritizes the recency of publication. Starting from 118 studies published since 1999, 88 review studies were identified based on the focus. Subsequently, after applying the procedure, 16 review studies were identified [20,21,22,23,24,25,26,27,28,29,30,31,32,33,34,35].

## 3. Results

In total, 16 reviews were selected [20,21,22,23,24,25,26,27,28,29,30,31,32,33,34,35].

The results are systematically organized into four sections, aligning first with the general objective and then with the specific aims in more detail.

Section 3.1 offers a comprehensive overview of how the subsequent sections of the results are structured. It highlights the key themes and topics that will be addressed, providing readers with a roadmap of the analysis. By outlining the organization, Section 3.1 ensures clarity and sets expectations for the flow of the results, guiding the reader through the various aspects and challenges related to ocular neuroprosthetics, from technical advancements to ethical considerations and future directions. This helps to contextualize the detailed exploration in the following sections, ensuring a coherent understanding of the broader subject matter.

Section 3.2 addresses the first objective by presenting trends observed across the studies, enhanced by graphical representations that visually illustrate the data. This section aims to highlight overarching patterns and significant shifts within the field, providing a clear view of how the research landscape has evolved over time.

Section 3.3 aligns with the second specific objective and includes: (a) Extraction of common themes and interconnections among the studies, highlighting recurring messages and drawing correlations between research findings. This synthesis allows for broader implications to be understood within the literature. (b) A detailed categorization of the studies, distinguishing fine-grained thematic areas as well as broader categories. This categorization organizes the research into specific subfields and general domains, offering a comprehensive view of the diverse aspects covered and pinpointing key areas of focus and specialization.

Section 3.4 answers the third aim and identifies opportunities and areas requiring further research exploration. This section sheds light on areas of emerging interest that warrant deeper investigation.

### 3.1. Synoptic Overview of the Organization of the Results

**Figure 2:**
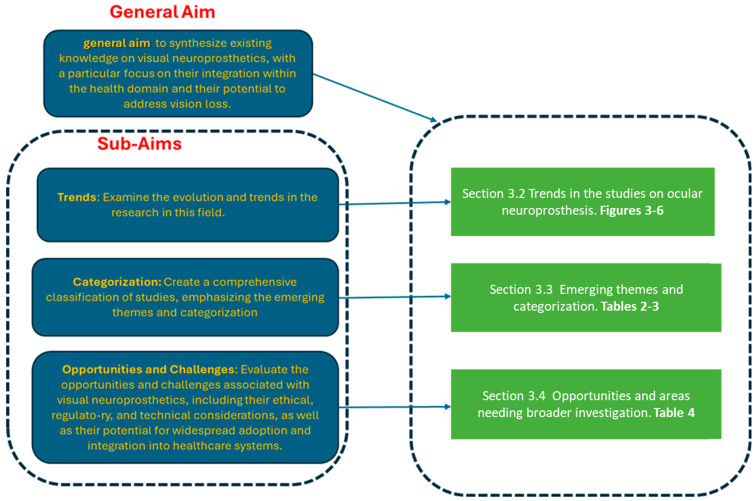
Synoptic diagram of the organization of the results.

The synoptic diagram in Figure 2 provides a comprehensive visualization of the organization and flow of the rationale underpinning the presentation of results, ensuring alignment with the overall objectives of the study. On the right-hand side, moving from top to bottom, the diagram is divided into three main blocks.

**Figure 3:**
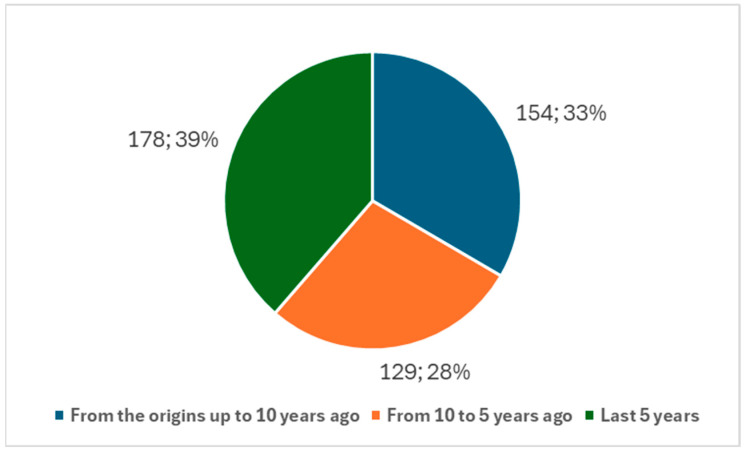
Trends in the number of publications on ocular NP over the last five years, the last ten years, and earlier.

**Figure 4:**
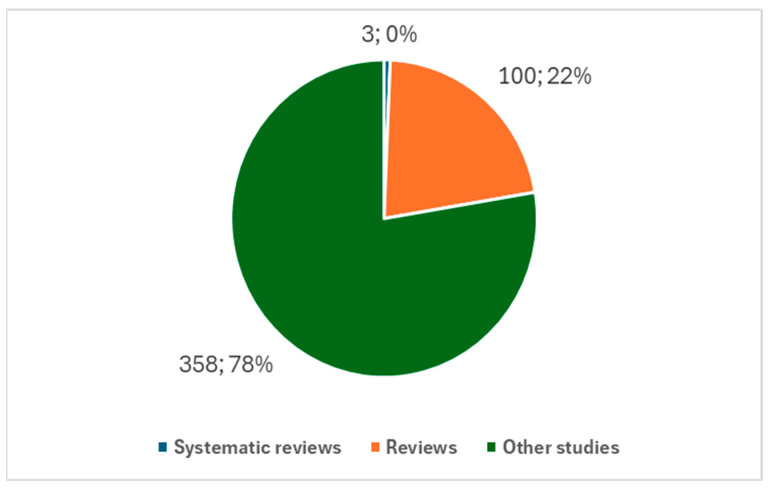
Distribution of the different types of studies.

**Figure 5:**
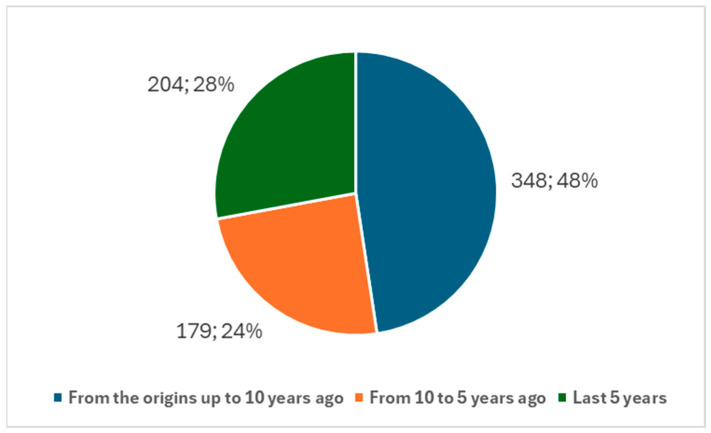
Trends in the number of publications on NP over the last five years, the last ten years, and earlier.

**Figure 6:**
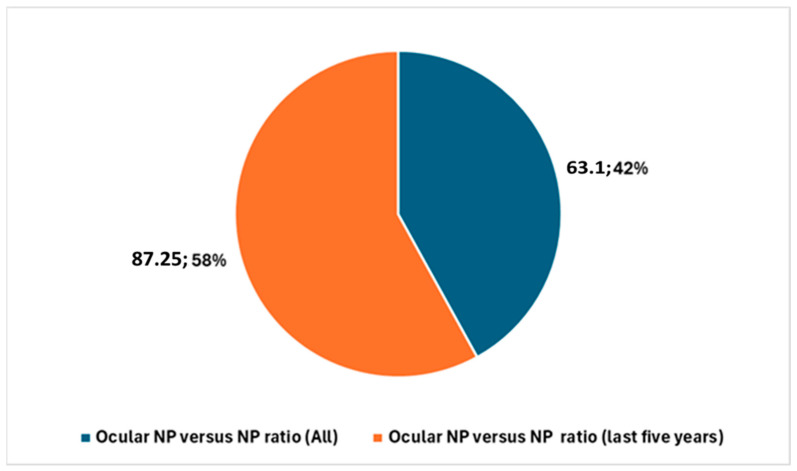
Ratio of publications on the ocular neuroprosthesis to total neuroprosthesis publications, with a focus on the last five years.

The first block highlights bibliometric trends, as discussed in Section 3.2, offering insights into the evolution and patterns within the literature. These trends are further substantiated by Figure 3, Figure 4, Figure 5 and Figure 6, which provide visual representations and detailed data analysis. This section aligns with the study’s first sub-aim, focusing on establishing a foundational understanding of the research landscape.

The second block focuses on emerging themes and their systematic categorization, as detailed in Section 3.3. These themes represent the key areas of discussion identified during the review process. The insights are supported by Table 2 and Table 3, which present detailed breakdowns and classifications of these themes to enhance clarity and depth of understanding.

Finally, the third block addresses the opportunities for further research and identifies areas that require expansion and deeper investigation. This is elaborated in Section 3.4, where specific gaps and potential avenues for future work are discussed. The findings in this section are corroborated by Table 4, which provides a structured summary of these opportunities and recommendations for future exploration. Together, these components form a coherent and structured presentation of the study’s findings.

### 3.2. The Trends in the Studies on Ocular Neuroprostesis

A PubMed search highlights the trends in the evolution of publications in the field of neuroprosthesis research, focusing on the ocular neuroprosthesis. For this analysis, keyword 1 from Box 1 was used to identify studies specifically related to the ocular neuroprosthesis.

Since 1971, a total of 461 studies on the ocular neuroprosthesis have been published. Over time, there has been a noticeable increase in research activity. Specifically, in the last 10 years, 307 studies were published, representing 66.59% of the total publications on the ocular neuroprosthesis (Figure 3). Narrowing this period even further, in the last five years, we find 178 studies, which account for 38.61% of the total publications (Figure 3).

This growth highlights the increasing attention and relevance of the ocular neuroprosthesis within the broader field of neuroprosthesis research.

A closer examination of the types of studies reveals that systematic reviews are rare in this domain. Out of the 461 studies on the ocular neuroprosthesis, only three systematic reviews have been conducted, while there are a total of 103 reviews (Figure 4). This suggests a need for more systematic reviews to synthesize the growing body of evidence in this area.

When comparing research trends in the ocular neuroprosthesis with those in the broader field of neuroprosthesis, a similar analysis was performed using keyword 2 from Box 1 to identify studies related to the neuroprosthesis in general. The results indicate a broader but less focused growth trend. Since 1971, a total of 731 studies on the neuroprosthesis have been published. Over the last 10 years, 383 studies were published, representing 52.39% of the total. Narrowing the scope to the last five years, 204 studies were published, accounting for 27.90% of the total (Figure 5).

This slower growth in neuroprosthesis research over the last five and ten years contrasts sharply with the more rapid increase in ocular neuroprosthesis research, suggesting a shift in focus or prioritization within the field.

A particularly striking finding is the proportion of neuroprosthesis studies that focus specifically on the ocular neuroprosthesis (NP). Across the entire period from 1971 to the present, studies on the ocular neuroprosthesis constitute 63.1% of all neuroprosthesis-related publications. When narrowing the analysis to the last five years, this percentage rises significantly to 87.25% (Figure 6). This increase underscores the growing importance of the ocular neuroprosthesis as a subfield of neuroprosthesis research, possibly reflecting advances in technology, greater interest in addressing vision-related disabilities, or the emergence of promising results in this domain.

Box 1 **The proposed composite keys.**

(ocular neuroprosthesis[Title/Abstract]) OR (Retinal implants[Title/Abstract]) OR (Bionic eyes[Title/Abstract]) OR (Vision prosthetics[Title/Abstract]) OR (Artificial retina[Title/Abstract]) OR (Optical neural implants[Title/Abstract])(neuroprosthesis[Title/Abstract])

Overall, this analysis highlights a clear trend: the ocular neuroprosthesis has become a focal point within the broader field of neuroprosthesis research, with a faster growth rate in publications and a steadily increasing share of the total body of work. However, the limited number of systematic reviews emphasizes the need for a more structured synthesis of evidence to guide future research and clinical applications.

### 3.3. Emerging Themes and Categorization

Recent advancements in visual restoration have brought about profound changes in the field of vision science, offering hope for individuals suffering from degenerative retinal diseases. These technologies encompass a variety of strategies, from retinal prosthetics to gene therapies and biotechnological innovations, each aiming to either restore or replace lost vision. From the review overview, multifaceted and highly functional studies in this field emerge [20,21,22,23,24,25,26,27,28,29,30,31,32,33,34,35].

Among the most established solutions, retinal prostheses such as the ARGUS II system have shown promise in restoring basic vision in patients with retinal degeneration by electrically stimulating surviving retinal ganglion cells (RGCs) to bypass damaged photoreceptors. This system enables patients to perceive light, motion, and basic shapes, though its resolution remains limited due to factors such as current spread in retinal tissue, which limits spatial precision and image quality [20,21,33]. While these prosthetics offer vital improvements in quality of life, the need for higher resolution and more complex visual experiences has led to the exploration of alternative and complementary approaches.

Cortical prostheses, which bypass the retina entirely by stimulating the visual cortex, have emerged as an alternative, particularly for individuals with advanced retinal degeneration. These systems offer greater flexibility since they do not rely on the preservation of any retinal structures. By directly stimulating the brain’s visual processing centers, cortical prostheses aim to restore more complex visual perceptions. However, challenges persist in achieving high visual acuity and producing the nuanced visual experiences that are typically processed by the retina. Recent advances suggest that using multiple channels of cortical stimulation may enhance the depth and complexity of the visual experience [20,24,33].

Building on these technologies, hybrid systems are being explored, combining both retinal and cortical stimulation to enhance visual function. These hybrid systems aim to address the limitations of each individual approach by utilizing the strengths of both retinal ganglion cell stimulation and cortical stimulation, potentially offering a more comprehensive solution for visual restoration [23,31].

In parallel, regenerative prostheses that focus on biological repair or replacement of damaged retinal cells are gaining attention. This approach includes gene therapies and nanotechnology, both of which aim to address the underlying causes of degenerative diseases at the molecular level. Gene therapies, for instance, aim to restore the function of photoreceptors by introducing functional genes into the retina, offering the potential for halting disease progression and, in some cases, partially restoring sight. Meanwhile, nanoparticles are being developed to target specific retinal cells with high precision, providing less invasive methods for stimulation and potentially enhancing the effectiveness of visual prosthetics [23,34,35].

Additionally, non-invasive stimulation techniques are gaining momentum as they offer a way to restore vision without the need for implants. These methods include transcranial magnetic stimulation (TMS) and optogenetic approaches, which aim to modulate visual processing through external stimulation of the brain or retina. Although these techniques are still in early stages of development, they present a promising avenue for those who may not be candidates for traditional prosthetic devices [24,28].

Collectively, these advancements reflect a rapidly evolving landscape in visual restoration. By combining hardware-based solutions with biological and non-invasive techniques, the goal of restoring or even surpassing natural vision capabilities is becoming increasingly attainable. As these technologies progress, they hold the potential to significantly improve the quality of life for individuals with vision loss, offering new possibilities for those affected by retinal degenerative diseases [20,23,35].

In an overview on ocular neuroprosthetics, it is crucial to include a diverse range of studies that not only concentrate on the development of prosthetic devices but also address foundational, technological, and interdisciplinary insights that inform their progress.

For example, while Fieldman’s philosophical examination of vision challenges the reductionist theories of visual perception [20], it provides valuable context for understanding the theoretical limits and ethical considerations in the design of visual prosthetics. Additionally, Kurbis et al. [25] contribute by exploring AI-based navigation systems in robotic prosthetics, emphasizing the growing intersection of robotics and human-centered design. This approach is vital for understanding the practical application of neuroprosthetics in everyday life, such as mobility and interaction.

Furthermore, the inclusion of studies like Wang et al. [21], which explores the use of advanced materials like negative photoconductivity transistors for artificial vision, is essential for highlighting the technological innovations that underpin the development of neuroprosthetic devices. These insights are not only valuable for engineers and scientists working on device improvement but also for clinicians and patients who benefit from the ongoing advancements in neuroprosthetic functionality.

By including diverse perspectives—ranging from theoretical discussions on the neural binding problem [20] to practical advancements in prosthetic AI navigation [25] and material innovation [21]—a comprehensive review can offer a holistic understanding of the current landscape of neuroprosthetics. This approach underscores the importance of interdisciplinary collaboration in overcoming challenges and shaping the future of vision restoration and sensory prosthetics. It allows for a more nuanced discussion that integrates ethical, technical, and practical considerations, fostering a broader appreciation of the complexity involved in the development of these life-changing technologies.

Table 2 reports a specific categorization with a brief description of each contribution and the focus.

This table serves as a comprehensive reference to significant advancements in the fields of vision restoration, retinal disease management, and prosthetics. These advancements span a wide array of approaches—from neural interfaces and artificial retinas to gene therapies, nanotechnology, and bioprinting. Below, we expand on each theme while introducing key references to illustrate the breadth of ongoing research and technological developments.

Philosophical and Theoretical Foundations of Vision

The exploration of fundamental questions about human vision and perception remains a cornerstone of vision science. Fieldman [20] addresses the neural binding problem and subjective visual experience, emphasizing that current reductionist theories fall short of fully explaining the complexities of human vision. This philosophical inquiry underscores the need for scientific breakthroughs that can address deep-seated mysteries of how vision works and how prosthetics might replicate or restore it.

Artificial Retina and Neural Interface Technologies

Advancements in artificial retina technologies are rapidly progressing through the use of specialized materials that can replicate aspects of biological vision. Wang et al. [21] delve into visuomorphic computing, a concept that utilizes negative photoconductivity transistors (NPTs) to simulate biological vision through optoelectronic devices. These materials not only promise improvements in artificial vision but also open pathways for optoelectronic logic gates and multibit memory systems. Similarly, Pang et al. [30] discuss the promising applications of graphene in artificial retinas and tactile sensors, noting its potential for neuromorphic computing and artificial muscles that could interact with sensory interfaces to aid in visual restoration.

Gene and Cell-Based Therapies for Retinal Diseases

Recent breakthroughs in precision medicine have provided hope for patients suffering from inherited retinal diseases (IRDs) like Retinitis Pigmentosa (RP). Girach et al. [32] emphasize the transformative potential of gene editing, optogenetics, and RNA-based therapies, particularly focusing on novel strategies like antisense oligonucleotides. This aligns with Kamde and Anjankar’s [27] findings on the use of gene and cell therapies for restoring vision in individuals with RP. These treatments are still in their early stages, but they represent a promising direction for curing or significantly alleviating the effects of inherited retinal disorders.

Nanotechnology and Light-Sensitive Materials for Vision Restoration

Nanotechnology continues to play a central role in advancing retinal prosthetics. Stoddart et al. [23] highlight the development of nanoparticle-based optical transduction systems, which enable precise retinal stimulation with improved resolution and reduced invasiveness. Their work focuses on light-sensitive nanoparticles that can target retinal ganglion cells more efficiently, a critical step in restoring sight. Additionally, Zaho et al. [31] explore the use of photoactive nanomaterials for neural interfaces, discussing their potential for wireless brain stimulation and their challenges in maintaining long-term stability within the body.

Artificial Intelligence and Assistive Technologies

AI and machine learning are revolutionizing assistive technologies for individuals with visual impairments. Kurbis et al. [25] introduce the StairNet initiative, a deep learning platform designed to enhance the mobility of prosthetic users by improving real-time perception of stairs. This kind of human-centered design represents a significant leap in prosthetics that incorporates intelligent, adaptive systems. The integration of AI into visual prosthetics is not only improving mobility but also enhancing the overall user experience by enabling more natural interactions with prosthetic devices.

Retinal Prosthetics and Non-Invasive Brain Stimulation

Lestak [24] presents a non-invasive approach to ocular prosthetics, which involves transcranial stimulation of intact visual cortex cells. This represents a potential advancement in the treatment of blindness, as it reduces the risks associated with invasive electrode implantation. Moreover, Kim et al. [34] focus on enhancing neural signal transmission within retinal prosthetics. By using computational modeling to optimize the way retinal ganglion cells transmit visual information, they aim to improve the quality of artificial vision, making it more similar to natural sight.

Bioprinting and Tissue Engineering

Bioprinting has emerged as a groundbreaking approach to creating functional retinal tissues and prosthetics. Kravchenko et al. [28] explore various bioprinting techniques, such as extrusion and laser-assisted methods, which are being used to fabricate complex tissues like bioprinted corneas and retinal tissue engineering. The development of implantable electrode arrays is another exciting application, as it holds the promise of directly interfacing with the retina to restore sight with precision.

Age-Related Macular Degeneration (AMD) and Therapeutic Innovations

AMD continues to be a major area of focus in vision research, with significant advances in both pharmacologic treatments and innovative therapies. Borchert et al. [29] discuss emerging therapies such as complement pathway inhibitors (e.g., Pegcetacoplan) and the use of optogenetics for restoring vision. Meanwhile, Nanegrungsunk et al. [35] explore technological and pharmacological innovations that aim to reduce the treatment burden for AMD, such as long-acting anti-VEGF agents and gene therapy.

Interdisciplinary Approaches and the Future of Vision Restoration

The research and technological advancements highlighted in the table reflect the dynamic and multidisciplinary nature of modern vision restoration efforts. From philosophical reflections on visual perception [20] to cutting-edge materials like graphene for artificial retinas [30], these studies demonstrate a concerted push towards personalized therapies and innovative prosthetics that could fundamentally alter the landscape of vision science. As these fields continue to intersect, the possibility of achieving fully functional prosthetic vision and restoring sight becomes increasingly tangible.

The synergy between biotechnology, nanotechnology, AI, and gene therapy represents the future of vision restoration, with the potential not only to improve quality of life for individuals with visual impairments but also to radically shift the way we think about human–machine interfaces and the boundaries of medical science.

Table 3 presents the identified broad areas of interest.

**Table 2:** Sketch of the review with focus and.

Reference	Brief Description	Focus	Categorization
Fieldman [20]	Explores fundamental mysteries of human vision, including the neural binding problem, blind sight, and subjective experience, alongside advancements in prosthetics. Suggests that fundamental scientific breakthroughs are required to resolve core mysteries of the mind.	Neural mechanisms underlying vision, limitations of reductionist theories, and future scientific challenges.	Philosophical and neuroscientific insights into visual perception and prosthetics.
Wang et al. [21]	Reviews the concept of visuomorphic computing, simulating biological vision with artificial retina technologies like negative photoconductivity transistors (NPTs). Highlights applications such as optoelectronic logic gates and multibit memory, addressing challenges in energy efficiency and complexity reduction.	Advanced materials and devices for artificial vision, emphasizing NPT applications and future optoelectronic computing.	Artificial retina technologies and visuomorphic computing.
Kelly et al. [22]	Discusses advancements in biotic–abiotic interfaces, such as brain–computer interfaces and organic transistors, to restore lost neural functions. Covers technologies enabling communication between biological tissues and synthetic devices, emphasizing restoration of vision and motor abilities.	Biotic–abiotic interfacing, neural signaling restoration, and interdisciplinary approaches for sensory prosthetics.	Neural interface technologies for sensory restoration and prosthetic integration.
Stoddart et al. [23]	Reviews nanoparticle-mediated optical transduction for targeting retinal ganglion cells in visual prosthetics. Focuses on light-sensitive nanoparticles to reduce invasiveness and improve single-cell resolution in retinal stimulation.	Nanoparticle-based retinal stimulation, emphasizing pharmacokinetics and clinical translation.	Nanotechnology for retinal prosthetics and vision restoration.
Lestak [24]	Presents the evolution of cortical prostheses and introduces a non-invasive transcranial stimulation method targeting intact visual cortex cells. Highlights limitations of current systems, including neural damage from electrodes.	Non-invasive cortical stimulation methods, historical development of visual cortex prosthetics.	Visual cortical prostheses and non-invasive brain stimulation techniques.
Kurbis et al. [25]	Introduces the StairNet initiative, a deep learning platform for real-time visual perception of stairs, aiding robotic prosthetics and human–robot interaction. Highlights high classification accuracy and mobile deployment for prosthetic navigation.	AI-based navigation for robotic and prosthetic systems, focusing on human-centered design for mobility enhancement.	Artificial intelligence and visual perception for assistive robotics.
Papaionnou [26]	Explores age-related macular degeneration (AMD) therapies, including anti-VEGF agents, gene therapies, retinal implants, and AI algorithms for diagnosis. Highlights innovations like Pegcetacoplan and Faricimab for advanced AMD management.	AMD management, including pharmacologic treatments, gene therapies, and AI for monitoring and diagnosis.	Therapeutic advancements and AI in AMD treatment.
Kamde and Anjankar [27]	Discusses retinitis pigmentosa (RP), its genetic basis, and treatment options such as gene and cell therapies, retinal implants, and vision restoration strategies. Emphasizes ongoing research in genetic treatments and rehabilitation.	Gene therapies, rehabilitation, and future innovations in managing inherited retinal dystrophies.	Genetic and cell-based treatments for Retinitis Pigmentosa.
Kravchenko et al. [28]	Highlights bioprinting applications in ophthalmology, such as bioprinted corneas, retinal tissue engineering, and implantable electrode arrays for visual prosthetics. Reviews bioprinting technologies, including extrusion and laser-assisted methods.	Tissue engineering for ophthalmology and neuroprosthetics, with a focus on bioprinting techniques and materials.	Bioprinting for visual prostheses and ophthalmological applications.
Borchert et al. [29]	Examines therapeutic approaches for AMD, emphasizing inflammation control, optogenetics, and emerging treatments like complement inhibitors (Pegcetacoplan) and retinal implants.	AMD therapy innovations, focusing on molecular mechanisms and neuroinflammatory degeneration.	AMD-focused therapeutic strategies, including molecular and technological interventions.
Pang et al. [30]	Discusses biomedical applications of graphene, including artificial retinas, tactile sensors, and brain–machine interfaces for sensory restoration. Explores its potential in neuromorphic computing and artificial muscles.	Graphene-based technologies for artificial vision, neuromorphic computing, and sensory interface design.	Advanced materials for sensory restoration and brain–machine interfaces.
Zaho et al. [31]	Focuses on nanomaterials for neural interfaces, particularly photoactive materials for retinal prosthetics and wireless brain stimulation. Reviews challenges in achieving long-term stability and neural regeneration.	Nanotechnology for vision restoration, wireless stimulation, and neural tissue engineering.	Nanomaterials for neural interfaces and vision restoration.
Girach et al. [32]	Reviews advancements in precision medicine for inherited retinal diseases (IRDs), including gene editing, optogenetics, and RNA-based therapies. Highlights antisense oligonucleotides as a novel approach.	Precision medicine in IRDs, with emphasis on gene therapies and optogenetic approaches.	Genetic therapies and precision medicine for inherited retinal diseases.
Kravchenko et al. [33]	Compares retinal, optic nerve, and cortical prostheses, focusing on advancements in devices like ARGUS II. Highlights challenges in improving visual resolution and user experience.	Development and limitations of current visual prostheses, with a focus on future device improvements.	Visual prosthetic advancements and comparative evaluation.
Kim et al. [34]	Examines strategies for enhancing retinal prosthetics by improving neural information transmission in retinal ganglion cells. Reviews computational approaches to optimize signal transmission rates.	Computational modeling for improving artificial vision quality and retinal stimulation.	Neural optimization in retinal prosthetics for enhanced visual perception.
Nanegrungsunk et al. [35]	Highlights pharmacological and technological progress in retinal disease management, including long-acting anti-VEGF agents, gene therapy, and reduced treatment burdens through sustained delivery methods.	Advanced pharmacology and reduced treatment burden approaches for retinal diseases.	Technological and pharmacologic innovations in retinal disease management.

**Table 3:** Identified broad areas of interest.

Macro Area	Description	References
Philosophical and Theoretical Foundations of Vision	Addresses fundamental questions about human vision, neural binding, and subjective experience, highlighting the need for scientific breakthroughs to address vision’s complexities.	Fieldman [20]
Artificial Retina and Neural Interface Technologies	Discusses advancements in artificial retina technologies, including the use of negative photoconductivity transistors (NPTs) for simulating biological vision and optoelectronic devices.	Wang et al. [21], Kelly et al. [22]
Gene and Cell-Based Therapies for Retinal Diseases	Focuses on precision medicine for inherited retinal diseases, with an emphasis on gene editing, optogenetics, and RNA-based therapies.	Girach et al. [32], Kamde and Anjankar [27]
Nanotechnology and Light-Sensitive Materials for Vision Restoration	Covers the role of nanotechnology, particularly nanoparticle-based optical transduction and photoactive materials, in retinal prosthetics and vision restoration.	Stoddart et al. [23], Zaho et al. [31]
Artificial Intelligence and Assistive Technologies	AI-driven innovations like StairNet for enhancing mobility, human-centered design in prosthetics, and integrating AI to improve prosthetic function and user experience.	Kurbis et al. [25], Papaionnou [26]
Retinal Prosthetics and Non-Invasive Brain Stimulation	Explores advances in retinal prosthetics and non-invasive cortical stimulation methods for restoring sight.	Lestak [24], Kim et al. [34], Kravchenko et al. [33]
Bioprinting and Tissue Engineering	Investigates the use of bioprinting for creating functional retinal tissues and prosthetics, as well as implantable electrode arrays for visual prosthesis integration.	Kravchenko et al. [28]
Age-Related Macular Degeneration (AMD) and Therapeutic Innovations	Discusses emerging therapies for AMD, including pharmacologic treatments, optogenetics, and anti-VEGF agents, alongside advancements in reducing treatment burden.	Borchert et al. [29], Nanegrungsunk et al. [35]
Advanced Materials for Artificial Vision	Discusses biomedical applications of graphene, including its role in artificial retinas, tactile sensors, and brain–machine interfaces to aid in visual restoration.	Pang et al. [30]

### 3.4. Opportunities and Areas Needing Broader Investigation

The restoration of vision through neuroprosthetics and related technologies is an intricate and rapidly evolving field, encompassing diverse areas of study. Researchers are tackling challenges from the philosophical foundations of visual perception to cutting-edge developments in artificial retinas, gene therapies, and AI-enhanced prosthetics. This interdisciplinary approach has led to significant advancements but also exposes areas that require further exploration.

In this context, a variety of studies have emerged, offering both promising opportunities and highlighting the gaps that still need to be addressed. Philosophical inquiries, such as those presented by Fieldman [20], examine fundamental questions about visual experience and perception, providing a critical backdrop for the development of more sophisticated prosthetic devices. Meanwhile, the technical aspects of vision restoration have been explored through artificial retina technologies, as in Wang et al.’s study on negative photoconductivity transistors (NPTs) [21]) and through the use of nanotechnology for retinal stimulation, as seen in the work of Stoddart et al. [23] and Zaho et al. [31].

Gene and cell therapies also play a central role in restoring vision, with researchers like Kamde and Anjankar [27] and Girach et al. [32] focusing on innovative methods for tackling inherited retinal diseases, including gene editing and optogenetics. Moreover, AI-driven systems, such as Kurbis et al.’s StairNet initiative [25], have paved the way for improved prosthetics that enhance user experience and mobility by enabling real-time perception. In parallel, advances in bioprinting, as explored by Kravchenko et al. [28], open new avenues for personalized retinal tissue engineering.

Despite the significant progress, several key challenges remain, particularly in achieving long-term integration and stability of these advanced technologies. Issues such as optimizing energy efficiency in artificial retinas, improving the scalability of gene therapies, and ensuring the biocompatibility of nanomaterials require further research and development. Additionally, the complex neural dynamics involved in restoring vision through prosthetics, as noted by Kim et al. [34], remain an area of ongoing study. These challenges reflect the multifaceted nature of the field, which requires continuous interdisciplinary collaboration.

The following Table 4 summarizes these emerging opportunities and the critical areas where further research is needed.

**Table 4:** Emerging opportunities and areas needing a broader investigation.

Study	Emerging Opportunities	Areas Needing Further Research
Fieldman [20]	Highlights the potential for deeper understanding of neural binding and subjective experience, which could inform the development of more effective prosthetics.	The need for breakthroughs in explaining the complexities of human vision, especially in terms of neural mechanisms and subjective experience.
Wang et al. [21]	Advancements in negative photoconductivity transistors (NPTs) for simulating biological vision, offering applications in optoelectronic devices like logic gates and memory systems.	Further research is required to improve energy efficiency and scalability of NPTs for practical use in visual prosthetics.
Kelly et al. [22]	The potential for biotic–abiotic interfaces to restore vision and motor functions by bridging biological tissues with synthetic devices.	Exploration of optimal interface designs for seamless communication between biological and synthetic materials, along with improving long-term integration.
Stoddart et al. [23]	Nanoparticle-based optical transduction could offer less invasive, more precise retinal stimulation.	Overcoming challenges related to biocompatibility, stability, and effective integration of nanoparticles for long-term use.
Lestak [24]	Non-invasive cortical prosthetics, particularly transcranial stimulation, could offer an alternative to electrode-based approaches.	The effectiveness and safety of non-invasive stimulation techniques, including long-term impacts and user adaptation.
Kurbis et al. [25]	AI-driven systems like StairNet could improve real-time navigation for prosthetic users, enhancing mobility and user experience.	More research into the accuracy, adaptability, and real-time performance of AI-driven prosthetic systems in varied environments.
Papaionnou [26]	The integration of AI and machine learning with retinal implants offers the potential for improved diagnostic capabilities and individualized treatment plans for AMD.	Further exploration of AI’s role in detecting early AMD and optimizing the use of retinal implants in diverse patient groups.
Kamde and Anjankar [27]	Gene and cell therapies for retinal diseases like retinitis pigmentosa (RP) offer the possibility of restoring vision through genetic correction.	The need for large-scale clinical trials to validate the long-term efficacy of gene therapies for retinal diseases.
Kravchenko et al. [28]	Bioprinting could revolutionize tissue engineering, allowing for the fabrication of bioprinted corneas and retinal tissues for personalized prosthetics.	Research is needed to perfect bioprinting techniques for replicating complex ocular tissues and integrating them with prosthetic devices.
Borchert et al. [29]	Emerging therapies, such as complement inhibitors, alongside optogenetics, show promise in managing AMD and restoring vision in late-stage patients.	There is a need for more studies to understand the long-term outcomes of these therapies, including their ability to delay disease progression.
Pang et al. [30]	Graphene-based materials could be used for artificial retinas, tactile sensors, and neuromorphic computing, creating efficient sensory interfaces.	Research into the scalability of graphene-based materials for clinical applications, as well as long-term stability in biological environments.
Zaho et al. [31]	Photoactive nanomaterials may improve the effectiveness of retinal prosthetics, offering wireless stimulation capabilities.	The stability and longevity of these materials in neural interfaces, as well as their ability to regenerate or replace damaged retinal tissues.
Girach et al. [32]	Precision medicine techniques like gene editing and optogenetics hold promise for treating inherited retinal diseases (IRDs).	Continued investigation into optimizing genetic therapies and their delivery methods, as well as patient-specific treatment models.
Kravchenko et al. [33]	Comparison of retinal, optic nerve, and cortical prosthetics highlights the potential for new hybrid systems combining multiple approaches for improved vision restoration.	Further exploration of hybrid prosthetic systems to address limitations in visual resolution and expand functionality.
Kim et al. [34]	Computational modeling can enhance retinal prosthetic performance by optimizing neural signal transmission, potentially improving artificial vision quality.	More studies are needed to understand the complex neural dynamics involved and how to optimize prosthetic designs for individual patients.
Nanegrungsunk et al. [35]	Long-acting anti-VEGF agents and gene therapies offer ways to reduce the treatment burden for retinal diseases like AMD, improving patient outcomes.	The long-term effects and efficiency of sustained delivery systems for retinal disease treatments, as well as the integration of gene therapies with existing treatment protocols.

This table demonstrates the breadth of approaches being explored in vision restoration, ranging from philosophical inquiries to cutting-edge technologies like AI, nanotechnology, and bioprinting. It underscores the interdisciplinary nature of this research and highlights both the promising opportunities and the significant challenges that still need to be addressed. As these various fields converge, the future of vision restoration becomes increasingly promising, with personalized, targeted therapies offering hope for those with vision impairments.

## 4. Discussion

The discussion is arranged into 8 sections.

Section 4.1 offers a comprehensive overview of how the subsequent sections of the discussion are structured. It highlights the key themes and topics that will be addressed, providing readers with a roadmap of the analysis. By outlining the organization, Section 4.1 ensures clarity and sets expectations for the flow of the discussion, guiding the reader through the various aspects and challenges related to ocular neuroprosthetics, from technical advancements to ethical considerations and future directions. This helps to contextualize the detailed exploration in the following sections, ensuring a coherent understanding of the broader subject matter.

Section 4.2 outlines the evidence and contribution of the overview of reviews in light of the specific challenges posed by blindness, also contextualizing it with other technological contributions currently available. This section aims to position the review findings within the broader landscape of existing market growth predictions, solutions, and technological advancements, in particular assistive technologies and accessibility technologies.

Section 4.3 presents the direct and indirect recommendations emerging from the analysis of the studies. These recommendations are derived from the identified trends and patterns, offering insights for future developments and applications.

Section 4.4 complements the overview by highlighting cutting-edge research studies that are advancing in the directions identified by the themes in the review. This section showcases recent innovations and studies that are pushing the boundaries of the field.

Section 4.5 delves into the basis of recent cutting-edge studies into emerging ethical issues and certification implications in light of recent studies. It highlights the need for a balanced approach in evaluating ocular neuroprosthetic technologies, addressing concerns related to patient autonomy, privacy, and fair access, alongside the necessity for rigorous safety and efficacy certification standards.

Section 4.6 delves into the basis of recent cutting-edge studies into the emerging role of artificial intelligence (AI) in ocular neuroprosthetics, highlighting its potential to revolutionize the field. AI is increasingly being integrated into the development and optimization of neuroprosthetic devices, enabling enhanced pattern recognition, real-time adjustments, and personalized treatment plans for patients.

Section 4.7, based on recent studies, highlights the key challenges in ocular neuroprosthetics development, emphasizing the importance of energy efficiency, scalability, and biocompatibility. Energy efficiency is crucial for sustained device operation without frequent recharging; scalability ensures broader application to various patient needs, and biocompatibility is necessary to minimize adverse reactions and support safe integration with human tissue. Addressing these challenges is vital for the widespread success and long-term functionality of ocular neuroprosthetics.

Finally, Section 4.8 concludes by outlining the limitations of the study, providing a critical reflection on the scope of the review and areas that may require further exploration.

### 4.1. Synoptic Overview of the Organization of the Discusion

**Figure 7:**
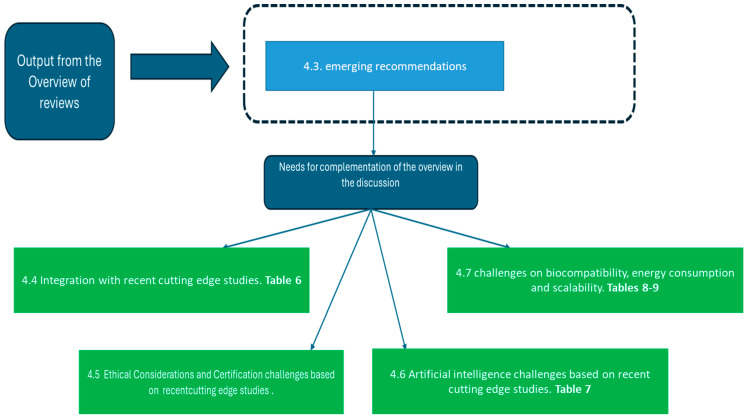
Synoptic diagram of the organization of the discussion.

The synoptic diagram in Figure 7 provides a detailed and structured visualization of the organization and flow of the rationale behind the presentation of the discussion, starting from the study results. The diagram is arranged into several key blocks, depicted on the right-hand side and moving sequentially from top to bottom.

The first block emphasizes the emerging recommendations derived from the review overview, as detailed in Section 4.3. These recommendations are supported by Table 5 and highlight the need to complement the existing review with additional analysis. This complementarity is elaborated in the subsequent blocks, which outline four further steps based on an in-depth analysis of recent cutting-edge studies in the field.

Moving from left to right and progressing downward, the diagram delineates the following key areas:

General Developments

The first block expands on the general advancements in the field, as discussed in Section 4.4. This section highlights the broader trends and developments, with Table 6 providing a comprehensive summary and corroborating the discussion.

Ethical and Certification Considerations

The second block, as detailed in Section 4.5, delves into the ethical challenges and certification processes, focusing specifically on critical issues and their implications for the field. This section underscores the importance of addressing these factors to ensure responsible and sustainable progress.

Integration with Artificial Intelligence

The third block, as detailed in Section 4.6, addresses the integration of artificial intelligence, a pivotal and rapidly evolving aspect of the field. This section discusses the opportunities and challenges associated with AI, supported by the detailed analysis presented in Table 7.

Scalability, Biocompatibility, and Energy Efficiency

The final block, as detailed in Section 4.7, focuses on significant challenges related to scalability, biocompatibility, and energy consumption. These factors are strategic for ensuring the long-term viability and functionality of prosthetic solutions. The analysis of these critical aspects is substantiated by Table 8 and Table 9, which provide data-driven insights and actionable recommendations.

In summary, the diagram in Figure 7 serves as a roadmap for the discussion, illustrating how each block builds upon the previous findings to address key themes, challenges, and opportunities in the field.

### 4.2. Highlights from the Overview of Reviews

#### 4.2.1. Added Values

To combat blindness in its various forms and ensure a good quality of life for people with this disability, there are established approaches that focus on different aspects of support and adaptation to individual needs.

The first key approach is improving the accessibility of living and working environments [36]. It is essential to create inclusive spaces that allow people with visual disabilities to live and work independently and safely. This includes physical modifications, such as the installation of tactile and sensory signage, but also the adoption of technologies that facilitate navigation and interaction with the surrounding spaces.

A second approach involves providing appropriate and personalized augmentative and alternative communication (AAC) tools [37]. These tools must be tailored to the individual’s specific needs. Blindness, in fact, presents different requirements depending on whether it is congenital (present from birth) or acquired after learning to read and write. In the case of congenital blindness, braille-based devices are much more suitable, while for those who lose their sight later in life or after literacy, these tools may be less useful. In these cases, AAC can include voice technologies or alternative visual input systems.

However, these approaches alone are not enough. It is necessary to integrate innovative regulatory tools, such as the ICF (International Classification of Functioning, Disability, and Health) [38,39], which allow for more precise customization of assistive technologies. The use of such systems enables providing solutions that adequately address the specific needs of users, optimizing the effectiveness of AAC in everyday and work contexts.

While AAC, possibly integrated with accessibility devices, allows information and communication to be provided in alternative modes using senses that are not impaired (such as hearing and touch), one of the most promising areas for the future is the restoration of visual function through neuroprosthetics. These advanced technologies aim to restore or improve visual perception by using devices that directly stimulate the brain circuits or ocular structures, partially or fully restoring the ability to see. Among neuroprosthetics, ocular devices are receiving particular attention, as highlighted by recent research and market growth forecasts.

In fact, there is expected to be significant growth in the demand for ocular prosthetics, with a rapidly expanding market that includes both ocular implants and optic nerve stimulation devices [18].

Our study provides added value in the field of ocular neuroprosthetics.

First, we have integrated a comprehensive overview of current technologies, from the ARGUS II system to cortical and hybrid prostheses, highlighting significant advancements as well as persistent challenges, such as limited resolution and the complexity of visual perceptions [20,21,33].

Second, we have explored regenerative approaches like gene therapies and nanotechnology, which are essential for addressing the underlying causes of retinal diseases and enhancing the effectiveness of visual prosthetics [23,34,35].

Additionally, our study incorporates an analysis of non-invasive techniques, such as transcranial magnetic stimulation (TMS), offering a new frontier for vision restoration without invasive implants [24,28].

Another key added value is the interdisciplinary approach that combines philosophical reflections on visual perception, such as those by Fieldman [20], with technological advancements like the use of innovative materials for simulating biological vision, such as negative photoconductivity transistors (NPTs) [21], and the application of artificial intelligence in prosthetic navigation systems [25]. This integrated approach allows for a more comprehensive understanding of ongoing progress and fosters more effective solutions.

#### 4.2.2. Expanded Considerations

Emerging technologies in ocular neuroprosthetics [9,10,11,12,13,14,15,16,17,18], as identified in this review, highlight significant advancements in restoring visual capabilities for individuals with varying degrees of vision impairment. Among these, non-invasive electrical stimulation devices [16,17] stand out as a promising, non-surgical option for enhancing visual functionality. These devices stimulate the optic nerve or other visual pathways externally, aiming to improve light perception, contrast sensitivity, and basic shape recognition. Studies suggest that these devices can enhance spatial awareness and assist in daily tasks for individuals with residual vision. However, their effectiveness varies significantly due to factors such as the specific area of stimulation, patient responsiveness, and the parameters of the applied stimulation. Despite these encouraging results, long-term efficacy, potential side effects, and sustained benefits remain areas requiring more robust clinical trials and exploration. These findings underline the need for patient-specific approaches in determining suitable candidates for this therapy, as not all individuals respond uniformly to non-invasive electrical stimulation.

In comparison, retinal prostheses [9,10], such as the Argus II and the PRIMA System, offer a more invasive but effective approach for patients with degenerative retinal conditions, including macular degeneration and retinitis pigmentosa. These devices bypass damaged photoreceptors by directly stimulating retinal ganglion cells, enabling users to perceive basic visual stimuli such as light, shapes, and motion. Such functionality can significantly enhance spatial navigation and mobility, providing patients with greater independence. However, these prostheses are limited by their resolution and reliance on residual retinal function. Patients with advanced retinal degeneration may experience diminished benefits, as the effectiveness of stimulation correlates closely with the integrity of remaining retinal cells. Moreover, the implantation process and device integration present further challenges, making patient selection and post-operative management crucial for successful outcomes.

For individuals with complete blindness due to optic nerve or cortical damage, cortical prostheses [11,12], such as the Orion System, present a groundbreaking alternative. By directly stimulating the brain’s visual cortex, these devices bypass the eye and optic nerve entirely, enabling users to perceive basic visual stimuli like light and shapes. This capability significantly enhances spatial orientation and environmental interaction for patients without functional retinal structures. However, cortical prostheses face similar limitations in resolution and signal processing complexity. Unlike retinal prostheses, these devices must rely on the brain’s interpretation of rudimentary electrical signals, making the restoration of high-resolution vision a persistent challenge. The advanced calibration and integration required further increase the technological demands of these devices.

When comparing retinal and cortical prostheses, significant differences emerge in their energy consumption and resolution. Retinal prostheses are typically more energy-efficient due to their localized stimulation of retinal ganglion cells. This contrasts with cortical prostheses, which require higher energy inputs to process visual information externally and stimulate the visual cortex. In terms of resolution, retinal prostheses generally perform better for patients with residual retinal function, providing basic visual acuity, whereas cortical prostheses cater to those with no functional retinal or optic nerve structures, albeit at a lower resolution.

This comparative analysis underscores the unique strengths and limitations of each approach. Retinal prostheses are more suitable for individuals with partial retinal function, offering localized restoration of vision, while cortical prostheses provide a critical solution for those with no remaining retinal or optic nerve function. Both technologies represent vital advancements in addressing vision loss and blindness, offering hope to patients and underscoring the importance of tailored approaches to treatment.

#### 4.2.3. The Growth and Importance of Ocular Neuroprosthetics

The field of ocular neuroprosthetics, as highlighted in the findings, represents a significant and rapidly evolving area of scientific research. Over recent years, this domain has experienced substantial growth in funding and market expansion, driven by increasing interest and investment in innovative technologies designed to restore vision. This upward trend underscores the importance of ocular neuroprosthetics in addressing critical unmet needs in visual impairment and blindness, positioning it as a key focus within the broader landscape of neuroprosthetic research and development. The global neuroprosthetics market, encompassing ocular devices, has been experiencing substantial growth. In 2023, the market was valued at approximately USD 12.69 billion and is projected to reach USD 38.93 billion by 2032, exhibiting a compound annual growth rate (CAGR) of 13.28% during the forecast period [39]. Specifically focusing on ocular prostheses, the market was estimated at USD 1.5 billion in 2023 and is anticipated to grow to USD 2.3 billion by 2030, with a CAGR of 6.5% [18,40]. Significant government funding has been directed toward advancing ocular neuroprosthetics and related technologies. For instance, in December 2024, the Advanced Research Projects Agency for Health (ARPA-H) awarded up to USD 56 million to a multi-institutional team, including researchers from Northwestern University, to develop and test strategies for whole-eye transplants aimed at restoring vision [41]. Similarly, a federal funding agency awarded up to USD 47 million to support high-impact research focused on making eye transplants a reality, underscoring the commitment to advancing ocular health technologies [42].

The upward trend in funding and market growth for ocular neuroprosthetics highlights the dynamic nature of this field. With continued investment, the development of innovative solutions aimed at restoring vision is poised to accelerate, offering hope to individuals affected by visual impairments.

Ocular neuroprosthetics, such as the Orion [43], Argus II [44], and PRIMA systems [45], represent pivotal advancements in the field of vision restoration, offering new hope for individuals with severe visual impairments. These innovative technologies have garnered significant attention in recent years due to their potential to partially restore vision and improve quality of life for patients.

The Orion System, developed by Second Sight Medical Products, is a cortical prosthesis that bypasses the eye and optic nerve to stimulate the visual cortex directly. Designed for individuals with total blindness due to damage to the optic nerve or other structural issues, the Orion System has demonstrated its ability to enhance spatial awareness and basic visual perception. Early trials have shown that users can detect light, shapes, and motion, improving their ability to navigate their environments.

The Argus II, another innovation from Second Sight, specifically targets individuals with retinitis pigmentosa, a degenerative retinal condition. By electrically stimulating the remaining functional retinal cells, the Argus II enables patients to regain limited visual abilities, such as recognizing light and basic shapes. The Argus II has set a foundation for wider acceptance and development of retinal prosthetic technologies. The PRIMA System, developed by Pixium Vision, employs cutting-edge photovoltaic technology to address vision loss associated with atrophic age-related macular degeneration (AMD). Implanted sub-retinally, this device converts light into electrical signals to stimulate the retina. The PRIMA System’s minimally invasive design and integration with external augmented reality devices highlight its potential as a next-generation solution for AMD patients.

These technologies collectively reflect the progress made in ocular neuroprosthetics. While challenges remain—such as improving resolution, reducing invasiveness, and expanding accessibility—their development underscores a growing global commitment to addressing vision impairments through innovative and multidisciplinary approaches.

Rehabilitation pathways are also an essential component in maximizing the benefits of ocular neuroprosthetics. These interventions bridge the gap between the technological advancements of devices and the patient’s ability to effectively integrate them into daily life. The importance of tailored rehabilitation programs is underscored by their ability to optimize outcomes, improve quality of life, and support long-term adaptability for individuals with visual impairments.

A strategic and essential component in the success of neuroprosthetic interventions is the rehabilitation process following the implantation. This phase plays a pivotal role in helping patients adapt to and optimize the functionality of the implanted device. Effective rehabilitation programs are tailored to the individual, focusing on improving sensory perception, enhancing neural plasticity, and ensuring the seamless integration of the device into the patient’s daily life. These efforts not only maximize the potential benefits of the neuroprosthetic but also significantly contribute to the patient’s overall quality of life and independence.

Neuro-optometric rehabilitation therapy plays a pivotal role in this process. As highlighted by [46], it focuses on retraining the visual system through structured activities and targeted exercises. This therapy is instrumental in enhancing visual processing and sensory-motor integration, particularly for patients adjusting to new sensory inputs provided by prosthetic devices. Similarly, [47] emphasizes that such rehabilitation pathways are vital for addressing the unique visual challenges faced by patients with neurological impairments, helping them regain confidence in navigating their environments.

The integration of neuroprosthetics into patient care also requires a multidisciplinary approach, as noted in [48]. The collaboration between engineering innovations and rehabilitative care ensures that the devices align with the complex needs of patients, enabling them to achieve functional improvements. Rehabilitation not only aids in adapting to prosthetics but also promotes neuroplasticity, which is crucial for the brain’s ability to interpret the electrical signals generated by these devices.

Finally, the emerging field of bionic connections, discussed in [49], highlights how neuroprosthetics establish a seamless link between the brain and external devices. Rehabilitation pathways facilitate this connection by helping patients refine their control over prosthetic systems, thereby improving sensory feedback and user experience.

By prioritizing structured rehabilitation strategies, healthcare providers can ensure that advancements in ocular neuroprosthetics translate into meaningful and sustainable improvements in the lives of individuals with visual impairments. Therefore, the rehabilitation market associated with neuroprosthetic technologies is also expected to experience significant growth. As the adoption of advanced neuroprosthetic devices increases, so does the demand for specialized rehabilitation services to support patients in adapting to these innovations. This trend underscores the importance of developing comprehensive rehabilitation programs and training professionals to meet the evolving needs of patients, creating new opportunities within the healthcare and medical technology sectors.

### 4.3. Emerging Recommendations from the Overview of Reviews

Our overview of reviews, particularly focused on the last three years, can give us an idea of the topics that have become well-established to the point where they can be further developed into reviews. On the other hand, it also offers strategic reflections based on the recommendations and challenges raised in those same reviews.

The development of neuroprosthetics, especially for vision restoration, represents a significant leap forward in medical technology. Devices like retinal implants, AI-enabled prostheses, and gene therapies hold promise for enhancing the lives of individuals with visual impairments. However, their successful integration into healthcare systems is not without its challenges. These challenges span across regulatory, ethical, and clinical dimensions, each requiring careful consideration to ensure the safe, effective, and equitable implementation of these technologies.

Regulatory Dimensions

Neuroprosthetic technologies, particularly those involving bioprinted retinal tissues or nanoparticle-based stimulators, are advancing at a rapid pace. However, current regulatory frameworks, such as those set by the FDA or EU Medical Device Regulation (MDR), often lag behind technological developments. These frameworks, while rigorous, were not originally designed to address the unique risks and benefits of emerging neuroprosthetic technologies.

To overcome this gap, adaptive regulatory guidelines should be established to address the novel challenges posed by advanced technologies like gene editing and AI-driven devices. Standardizing international regulations can facilitate faster and more efficient device approvals and cross-border clinical trials, enabling broader access to these life-changing technologies. Moreover, ongoing post-market surveillance is crucial to monitor the long-term safety and efficacy of these devices in real-world conditions, ensuring they continue to meet safety standards over time [23,24,28].

Ethical Dimensions

The ethical concerns surrounding neuroprosthetics are complex and multifaceted. One of the primary issues is ensuring informed consent, particularly with the integration of AI systems into medical devices. Patients must fully understand the risks, benefits, and limitations of the technologies they are using. As these devices become more sophisticated, providing clear and understandable information becomes a growing challenge.

In addition, data privacy is a significant ethical concern, especially with the amount of sensitive neural data generated by AI-enabled neuroprosthetics. It is essential to implement robust privacy safeguards to protect patient information. With the potential for data breaches, a strong ethical framework for the management of such data is necessary.

Equitable access is another pressing ethical issue. While these technologies offer significant improvements for patients, they often come with high costs, which may limit accessibility. Creating subsidy programs and public–private partnerships can help reduce these barriers, ensuring that these technologies are available to all individuals, regardless of socioeconomic status or geographic location [25,27,35].

Clinical Dimensions

Clinically, personalization is key in neuroprosthetics. Due to the variations in patient anatomy and disease profiles, each device must be tailored to meet the unique needs of the patient. For example, retinal prostheses may need to be adjusted for each patient based on the progression of their condition. This requires a high degree of customization and emphasizes the importance of interdisciplinary collaboration among engineers, clinicians, and geneticists to ensure the optimal outcome for each patient.

Training healthcare professionals is also critical. Clinicians must not only be trained to implant these devices but also to support patients in long-term device maintenance. User support systems are necessary to ensure that patients can manage any issues that arise and adjust to the new technology effectively.

The longevity of devices is another concern. Neuroprosthetics must be designed to be durable and reliable over extended periods. Modular devices or graphene-based prosthetics that can be upgraded as technology advances are ideal solutions to reduce maintenance costs and enhance long-term patient satisfaction [29,34,35].

Cross-Cutting Challenges

One of the major cross-cutting challenges is balancing innovation with ethical and practical considerations. As neuroprosthetics continue to evolve, there must be a concerted effort to ensure that these devices are safe, effective, and accessible for all patients. This will require collaboration across various fields, including regulatory bodies, scientists, ethicists, and patient advocacy groups.

Scalability and affordability are also critical issues. To ensure that these technologies reach as many patients as possible, public–private partnerships should be established to reduce costs and improve manufacturing efficiency. These efforts are essential for making neuroprosthetic technologies accessible to a wider population, including those in underserved areas [23,28,32].

Table 5 summarizes these emerging challenges and recommendations.

**Table 5:** Emerging challenges and recommendations.

Category	Challenges	Recommendations	References
Regulatory	Lack of global standardization in regulations.	Harmonize international regulations; develop adaptive guidelines for emerging technologies.	[23,24,25]
	Rapid innovation outpacing regulatory frameworks.	Establish flexible regulatory pathways for bioprinting, gene therapy, and AI devices.	[23,28,32]
Ethical	Informed consent and autonomy concerns with complex devices.	Implement transparent communication protocols emphasizing risks, benefits, and limitations.	[20,27,35]
	Data privacy and cybersecurity issues related to AI-enabled devices.	Strengthen data privacy laws and establish ethical frameworks for AI usage.	[25,30,35]
Clinical	Customization challenges for individual patients.	Invest in interdisciplinary research for personalized therapies and ergonomic designs.	[22,28,33]
	Limited expertise in handling advanced devices.	Provide specialized training for clinicians and establish comprehensive support systems.	[24,26,28]
	Device longevity and maintenance concerns.	Develop modular, upgradable designs and establish long-term support services.	[24,29,35]
Cross-Cutting	Balancing innovation with ethical and regulatory issues.	Foster collaboration between regulators, scientists, ethicists, and patients.	[23,28,32]
	Scalability and affordability of advanced devices.	Support public–private partnerships to create cost-effective solutions.	[25,27,33]

The development of neuroprosthetics for vision restoration is a groundbreaking step forward in medicine. However, the integration of these technologies into healthcare systems requires addressing complex regulatory, ethical, and clinical challenges. By fostering collaboration and interdisciplinary efforts and ensuring that patient safety and equitable access are prioritized, neuroprosthetics can continue to evolve into a transformative tool for individuals with visual impairments.

### 4.4. Bridging Established Knowledge with Emerging Research

It is strategic to compare the outcomes of the overview of reviews with recent primary studies in the field that address emerging topics. This comparison is essential not only for understanding how the latest advancements in visual prosthetics relate to previous research but also to highlight how these innovations are taking shape in practical applications. In this context, 10 recent studies were selected that provide a continuum with the findings from earlier reviews, addressing issues such as usability, flexibility, and non-invasive methods of prosthetics.

The following Table 6 summarizes recent cutting-edge studies [50,51,52,53,54,55,56,57,58,59] in the field of visual prosthetics, highlighting both advancements and emerging technologies. These studies not only provide insight into the development of more effective and user-friendly devices but also reflect the progress made in improving visual restoration across a range of approaches, from AI-driven prosthetics to non-invasive stimulation techniques. These innovations align with ongoing efforts to refine the precision, adaptability, and comfort of visual prostheses, aiming for greater integration into everyday life and enhancing the overall quality of life for individuals with visual impairments. The table outlines key emerging cutting-edge research in the field of visual prosthetics, providing a comprehensive view of recent advancements.

User-centered design is prioritized by Nadolskis et al. (2024) [50], who emphasize the importance of usability, comfort, and real-world needs for prosthetic users. Their research shows that users tend to rely less on current implants than anticipated due to issues with usability and reliability. Improvements such as enhanced visual resolution, better integration with smart technologies, and greater independence in daily tasks are desired by users, highlighting the need for a participatory design approach.

In the domain of intelligent prosthetics, Liang et al. (2024) [51] explore AI and machine learning to enhance prosthetic functionality, such as object recognition and sensory integration. Their study demonstrates the effectiveness of strategies like salient object ranking (SaOR), which improves object identification and understanding in simulated prosthetic environments. Combining image descriptions with auditory feedback has led to significant progress toward more intelligent, context-aware vision systems.

On the front of non-invasive stimulation, Song et al. (2024) [55] have introduced temporal interference stimulation (TIS), a method that selectively stimulates retinal ganglion cells without the need for invasive implants. TIS achieves high spatial selectivity and reduces activation thresholds, providing an effective and safer alternative to conventional prosthetic systems.

Chemical neuromodulation is further explored by Vacca et al. (2024) [52], who discuss the use of solid-state nanopores for neurotransmitter delivery at the nanoscale. Their approach enhances the precision of neural activation, offering a promising avenue for developing more compatible and precise neural prosthetics.

Expanding the visual field of prosthetics is the focus of Hinrichs et al. (2024) [53], who have shown that prosthetics with wider visual fields (e.g., 45°) significantly improve navigation and environmental awareness. Their findings suggest that larger visual angles aid in more efficient exploration, faster learning, and better spatial awareness, which are essential for daily activities.

In the area of flexible materials, Zhu et al. (2024) [57] explore the development of flexible, biocompatible materials for prosthetics. Their research highlights materials like BiFeO3-BaTiO3 that exhibit strong photoelectric responses and offer potential for restoring vision in animal models, underscoring the importance of durable, adaptable artificial retinas.

Event-based imaging, introduced by Al Mahfuz et al. (2024) [58], uses quantum dot-enhanced circuits to simulate retinal amacrine cells, enabling transient responses to changes in light intensity. This novel approach eliminates traditional frame-based imaging, allowing for real-time, energy-efficient vision processing that is particularly useful in dynamic environments.

Retinal and cortical implants are further developed by Fernandez & Robles (2024) [54], who discuss the advancements in both retinal and cortical implants to restore vision at different levels of the visual processing system. These implants aim to address challenges such as electrode integration, biocompatibility, and long-term functionality, making them applicable to a wide range of visual impairments.

Improved electrical stimulation techniques, explored by Han et al. (2024) [56], focus on increasing the precision, energy efficiency, and control of visual prosthetic systems. Their use of Si nanowires and switched capacitor circuits enables more accurate and stable electrical stimulation, which is crucial for mimicking retinal responses effectively.

Lastly, the work of Jensen et al. (2024) [59] on simulation and modeling is helping accelerate the development of high-resolution prosthetics. By employing sparse and low-rank matrix approximations, their research has enabled a 10- to 133-fold acceleration in the simulation of multi-electrode arrays, making it more feasible to design large-scale electrode arrays for practical applications.

Together, these studies reflect a broad, interdisciplinary effort to improve visual prosthetics, advancing both the underlying technologies and their real-world application, from user-centered design to sophisticated neural stimulation methods.

Table 6 reports a sketch of the finding.

**Table 6:** Emerging research in cutting-edge studies.

Emerging Theme	Description	Key Findings	References
User-Centered Design	Prioritizing the real-world usability, comfort, and needs of individuals using visual prosthetics, aiming for devices that fit seamlessly into daily life.	Implantees often rely less on current implants than researchers anticipate, due to usability and reliability issues. Desired improvements include enhanced visual resolution, integration with smart technologies, and independence in daily tasks. These findings indicate a critical need for participatory design approaches.	Nadolskis et al. (2024) [50]
Intelligent Prosthetics	Leveraging AI and machine learning to enhance functionality, such as object recognition and sensory integration, for more natural and effective vision restoration.	AI-driven strategies like salient object ranking (SaOR) improve object identification and contextual understanding under simulated prosthetic vision. Coupling image description with auditory feedback enhances users’ cognitive and functional interactions in real-world scenarios, marking significant progress toward intelligent vision systems.	Liang et al. (2024) [51]
Non-Invasive Stimulation	Exploring low-invasive methods to stimulate retinal ganglion cells, avoiding risks associated with surgical implants.	Temporal interference stimulation (TIS) is demonstrated as a viable method for focused and selective retinal stimulation. The technique achieves high spatial selectivity and reduces thresholds for retinal ganglion cell activation, offering a promising alternative to invasive prosthetic systems.	Song et al. (2024) [55]
Chemical Neuromodulation	Introducing biomimetic chemical stimulation for precise and localized neural activation, improving interaction with neuronal circuits.	Solid-state nanopores enable neurotransmitter delivery at the nanoscale, offering higher spatial resolution and emulating synaptic activity. This approach has demonstrated effective neuronal stimulation in preclinical models, paving the way for chemically mediated neural prosthetics with better compatibility and precision.	Vacca et al. (2024) [52]
Enhanced Visual Field	Expanding the functional visual field of prosthetic devices to improve navigation, environmental awareness, and user safety.	Wider visual fields (e.g., 45°) significantly enhance navigation and task performance in naturalistic environments. Participants exhibited more efficient visual exploration, faster learning, and better spatial awareness, emphasizing the benefits of larger visual angles for daily activities.	Hinrichs et al. (2024) [53]
Flexible Materials	Developing flexible, biocompatible materials to integrate seamlessly with biological tissues, improving comfort and functionality of prosthetic devices.	Flexible composites of ferroelectric polymers and materials like BiFeO3-BaTiO3 exhibit strong photoelectric responses across a wide wavelength range. These materials demonstrate successful vision restoration in animal models, suggesting their potential for durable and adaptable artificial retinas.	Zhu et al. (2024) [57]
Event-Based Imaging	Creating event-driven imaging systems that mimic the transient responses of biological retinas, improving efficiency and real-time processing capabilities.	Quantum dot-enhanced circuits simulate retinal amacrine cells, providing transient spiking signals that respond to light intensity changes. These systems eliminate traditional frame-based imaging, offering real-time, energy-efficient vision processing suitable for dynamic environments.	Al Mahfuz et al. (2024) [58]
Retinal and Cortical Implants	Advancing both retinal and cortical implant technologies to restore vision at various levels of the visual processing system.	While retinal implants continue to improve, cortical prosthetics are emerging to overcome limitations related to retinal degeneration. Challenges include electrode integration, biocompatibility, and maintaining long-term functionality. This dual focus reflects the effort to address diverse types of blindness with tailored solutions.	Fernandez & Robles (2024) [54]
Improved Electrical Stimulation	Refining electrical stimulation methods for higher precision, energy efficiency, and control in visual prosthetic systems.	Advanced electrical stimulation techniques, such as those employing Si nanowires and switched capacitor circuits, allow for more precise and customizable current control. These systems show improved stability and accuracy in mimicking retinal responses, holding promise for applications requiring fine-tuned stimulation.	Han et al. (2024) [56]
Simulation and Modeling	Utilizing advanced computational techniques to accelerate the development and testing of high-resolution prosthetics, reducing time and cost.	Sparse and low-rank matrix approximations have enabled a 10- to 133-fold acceleration in the simulation of multi-electrode arrays. This innovation supports the iterative design of complex prosthetic systems, making large-scale electrode arrays more feasible for practical applications.	Jensen et al. (2024) [59]

Overall, these primary studies integrating the overview of reviews [50,51,52,53,54,55,56,57,58,59] go in the direction of both technological and methodological advancements as launched forward by the reviews analyzed:

1. Turning Theory into Practice

While studies like Fieldman [20] and Kim [34] emphasize the theoretical understanding of neural mechanisms and computational modeling, research such as [50] (user-centered design) and [59] (simulation and modeling) goes in the direction of attempting to bridge the gap by applying these concepts to real-world prosthetic designs. These advancements result in intuitive and effective devices tailored to user needs, ensuring that theoretical insights are grounded in practical utility.

2. Material Innovation and Scalability

Primary studies, such as Stoddart [23] and Zaho [31], highlight the potential of nanomaterials and nanoparticles in retinal prosthetics. The contributions of studies like [57] (flexible materials) and [58] (event-based imaging) refine these ideas, demonstrating biocompatible, scalable solutions that enhance both the functionality and user experience of prosthetic devices. Flexible ferroelectric composites and quantum dot-enhanced circuits directly address issues of stability and performance under dynamic real-world conditions.

3. Advancing Non-Invasive Technologies

Research into non-invasive cortical prosthetics (Lestak [24]) sets the stage for the breakthroughs in [45] (non-invasive stimulation). Temporal interference stimulation (TIS) expands the possibilities for retinal ganglion cell activation without surgical risks, offering practical alternatives to electrode-based systems discussed in earlier studies.

4. Artificial Intelligence Integration

Studies like Papaionnou [26] and Kurbis [25] explore AI’s potential to enhance prosthetic functionality, particularly for navigation and diagnostic tasks. The work in [51] (AI-driven intelligent prosthetics) builds on this foundation, demonstrating real-world applications such as salient object ranking and image description technologies. These advancements improve cognitive and functional interactions, making prosthetics smarter and more adaptive.

5. Enhanced Vision and Hybrid Approaches

Expanding the visual field [43] and integrating hybrid prosthetic systems [54] directly address the challenges identified by Kurbis [25] and Kravchenko [33]. Proposing solutions for overcoming the limitations of narrow visual angles and unidimensional approaches, these innovations provide a rich and the naturalistic visual experience for users.

6. Biotechnology and Neuromodulation

In line with the gene and cell therapy explorations in Kamde and Anjankar [27], studies such as [52] (chemical neuromodulation) and [56] (improved electrical stimulation) offer novel techniques for precise and localized neural activation. These advancements introduce biomimetic approaches that enhance compatibility and effectiveness compared to earlier methods.

7. Converging Biology and Engineering

Biotic–abiotic interfaces (Kelly [22]) and optogenetics (Girach [32]) lay the groundwork for the breakthroughs seen in [58] (simulation of amacrine cell circuits) and [54] (cortical and retinal implants). These studies not only deepen the integration of biology and synthetic systems but also make such interfaces more robust and scalable for clinical use.

The studies reported in [50,51,52,53,54,55,56,57,58,59] go in the direction of the increasing of the findings recorded in the review [20,21,22,23,24,25,26,27,28,29,30,31,32,33,34,35] by delivering practical, scalable solutions to the challenges identified in earlier research. These advancements refine theoretical insights into actionable technologies, ensuring an effective and user-centric approach to vision restoration and prosthetic design. By addressing gaps in scalability, usability, and biological integration, they represent a decisive step toward transforming visionary concepts into reality.

### 4.5. Ethical Considerations and Certification Challenges in Neuroprosthetic Advancements

The ethical and certification implications of visual neuroprostheses are multifaceted, encompassing concerns such as patient autonomy, equitable access, long-term safety, and the need for robust post-surgical support by manufacturers. Certification processes must address the challenges of ensuring device reliability, biocompatibility, and compliance with regulatory standards while fostering public trust. Additionally, there are broader ethical considerations, including the societal impact of such technologies, their potential to exacerbate inequalities, and the requirement for transparency and accountability in their development and deployment. These implications highlight the need for a comprehensive framework that integrates ethical, regulatory, and practical considerations to support the responsible advancement of ocular neuroprosthetics.

The ethical implications of visual neuroprostheses have been explored in a comprehensive review by Van Velthoven et al. [60], which identifies 169 ethical implications that have been categorized under seven main themes: (a) benefits for health and well-being; (b) harm and risk; (c) autonomy; (d) societal effects; (e) clinical research; (f) regulation and governance; and (g) involvement of experts, patients, and the public. These key themes are relevant to the ethical landscape of ocular neuroprosthetics, which share similarities with other neurotechnologies, such as brain–computer interfaces (BCIs) and cochlear implants (CIs).

One major theme is the benefits for health and well-being. Visual neuroprostheses offer significant potential for enhancing the quality of life in individuals with severe visual impairments, enabling better mobility and independence. However, the extent of the benefits, particularly in terms of restoring vision, remains a subject of ongoing ethical debate. Questions surrounding the realistic outcomes of these devices and their long-term impact on patients’ lives must be addressed to ensure that the benefits outweigh the risks.

Another prominent issue is harm and risk, as the use of ocular neuroprosthetics carries potential risks, such as device malfunction or physical harm due to the invasive nature of the implantation process. Ethical concerns include the balance between the possible harm from these devices and the potential benefits, as well as the extent to which patients should bear the risks associated with experimental technologies.

Autonomy is a central ethical principle, especially in the context of patient decision-making. For patients to make fully informed decisions about undergoing neuroprosthetic treatments, it is essential that they understand the risks, benefits, and limitations of these technologies. Ethical challenges arise if patients feel pressured to pursue treatment, especially when the long-term effects are uncertain or when the technology may not restore detailed vision.

The societal effects of visual neuroprostheses are another significant area of concern. These devices could influence broader social dynamics, including family relationships and community perceptions. Ethical questions arise about accessibility, where there may be inequalities in who can access these technologies and the potential societal pressures to undergo such treatments, which could affect patient autonomy and well-being.

The ethical considerations surrounding clinical research are also critical. Issues such as recruitment, informed consent, and long-term monitoring of participants are central to ensuring that clinical trials for visual neuroprostheses are conducted ethically. Rigorous ethical oversight is necessary to ensure patient safety and well-being throughout the course of clinical studies and beyond.

Regulation and governance are key to ensuring that visual neuroprostheses meet safety and efficacy standards before they are made available for clinical use. The review emphasizes the importance of ethical governance throughout the regulatory process, particularly regarding the post-market surveillance of devices to ensure their continued safety.

Lastly, the involvement of experts, patients, and the public is vital in the development of visual neuroprostheses. Input from diverse stakeholders ensures that the design, implementation, and regulation of these devices are aligned with the values and needs of those who will ultimately use them. Public engagement can also address societal concerns, promoting greater transparency and trust in the technology.

While there is significant literature on the ethical implications of other neuroprosthetic technologies, such as motor neuroprostheses and cochlear implants, a gap remains in the research specifically addressing the ethical issues of visual neuroprostheses. It is crucial that these ethical concerns are addressed early in the development process to ensure the responsible advancement of ocular neuroprosthetics, ultimately benefiting patients while minimizing risks and promoting fairness and equity.

Delving further into the regulatory aspects, regulatory pathways, the approval, and the regulation of ocular neuroprosthetics are highly complex processes that vary by region. In the United States, the FDA (Food and Drug Administration) plays a pivotal role in ensuring that devices meet rigorous safety and efficacy standards before being marketed to patients. For instance, the Argus II system underwent multiple stages of clinical trials and was approved by the FDA under the Humanitarian Device Exemption pathway [61], which facilitates the approval of devices intended for rare conditions. Similarly, in Europe, these devices must meet the standards set out by the CE marking process, which ensures that the product conforms to health, safety, and environmental protection standards. However, differing regulatory requirements across various regions can create discrepancies in how and when these technologies become available to patients, impacting the global accessibility of ocular neuroprosthetics. In some countries, regulatory frameworks may lag behind, leading to delays in the introduction of new technologies or more stringent regulations that could limit patient access.

A recent review, with particular focus also on the MD certification, identified 13 devices currently in use, categorized based on their stimulation location [62]. Six of these devices are actively involved in clinical trials, indicating ongoing research and development in the field of ocular neuroprosthetics. However, four devices—Alpha IMS, Alpha AMS, IRIS II, and ARGUS II—had previously received FDA and CE mark approvals but have since been discontinued. This discontinuation was largely influenced by factors such as evolving technological focuses and shifts in research priorities within the companies involved.

Of particular note is the ARGUS II, which stands out as a major achievement in the field. Despite receiving both FDA and CE mark approvals [63], which affirmed its safety and efficacy in clinical settings, the device was eventually discontinued. The company responsible for ARGUS II transitioned its focus toward visual cortical implants, demonstrating the rapidly evolving nature of research and development in ocular neuroprosthetics. This shift emphasizes that technologies in this field often evolve or are superseded by newer, more advanced solutions as research progresses.

The review also highlighted that while certain retinal prostheses have successfully demonstrated the ability to restore basic visual perception in both animal models and human patients, several challenges remain in bringing these devices to widespread clinical use. These challenges include the extended timeframes required to observe meaningful outcomes, the lack of standardized and validated measures for research, and the uncertainty surrounding the long-term effects of these devices. Additionally, limited funding and the small patient population—especially those diagnosed with retinitis pigmentosa who do not have comorbid conditions—pose significant barriers to the broader adoption and commercialization of these technologies.

Looking to the future, the review emphasizes that progress in retinal prosthetics should focus on developing more biocompatible, safe, and effective devices. Moreover, the exploration of visual cortical implants suggests that the field is moving toward even more sophisticated solutions for individuals with severe visual impairments. These advancements, alongside efforts to overcome the current challenges, could open new avenues for patients suffering from visual disorders that currently have few treatment options.

For the field of artificial vision and neuroprosthetics to advance, all of these factors highlight the critical importance of continuous support for patients and surgeons by the manufacturer of any implantable or integral device. Without such support, patients may be reluctant to participate in experimental procedures with implantable devices, potentially hindering overall progress in the field. The manufacturer’s role in providing long-term assistance—such as regular updates, technical support, and ensuring proper device maintenance—will be essential in building trust and fostering wider acceptance of these technologies. This ongoing support is crucial not only for patient safety but also for the sustained success and future development of ocular neuroprosthetics, ensuring that devices remain functional and effective over time while promoting greater confidence among patients and the medical community.

### 4.6. Integration with Artificial Intelligence Challenges in Neuroprosthetic Advancements

The integration of artificial intelligence (AI) in ocular neuroprosthetics represents a groundbreaking advancement in the field of vision restoration. While visual neuroprosthetic devices have already made strides in offering some level of visual perception to individuals with severe visual impairments, there remains significant potential for AI to enhance these technologies. AI-powered solutions are particularly poised to address some of the fundamental challenges that limit the effectiveness of current devices, including issues of image clarity, task-specific visual recognition, and the seamless integration of artificial vision into real-world activities. The future of retinal and visual prostheses lies in harnessing AI to not only improve basic vision restoration but also to enhance the quality of life for patients by enabling them to perform everyday tasks with greater ease and efficiency.

Recent studies have highlighted various AI innovations that could shape the next generation of ocular neuroprosthetics. One such innovation is the development of AI-driven visual augmentation, which shifts the focus from attempting to replicate natural vision to providing task-specific enhancements tailored to real-world needs. Beyeler and Sanchez-Garcia [64] proposed the concept of a smart bionic eye, which would provide visual augmentations powered by deep learning algorithms. Rather than simply trying to restore natural sight, this approach aims to enhance the ability to perform everyday activities such as face recognition, navigation, and self-care. AI’s ability to understand and prioritize visual information in real-time could make prosthetic vision more practical, offering a significant leap in usability for patients. Such augmentations would likely foster a greater sense of independence and confidence in visually impaired individuals, allowing them to navigate their environments and complete daily tasks with greater ease.

In addition to improving task-specific vision, AI is also being used to enhance the fundamental function of artificial retinas. One key challenge in retinal prosthetics is the difficulty in differentiating between significant and insignificant visual stimuli. Kim et al. [65] presented a solution to this problem by developing an artificial retina system that integrates artificial synapses with a signal-integration device, enabling attention-based signal processing. By filtering out irrelevant signals and focusing on important visual information, this AI-driven system mimics the human brain’s ability to prioritize certain stimuli over others. Such a system would enhance the device’s effectiveness by allowing users to focus on critical objects or tasks, such as recognizing faces in a crowd or identifying obstacles while walking, without being overwhelmed by unnecessary visual noise.

Another significant advancement in AI-enhanced retinal prosthetics involves optimizing image processing capabilities. Mehmood et al. [66] addressed a key limitation of current artificial retinas: the challenge of identifying multiple objects in a scene. Traditional image enhancement techniques, such as edge detection, are often insufficient in complex environments where numerous objects compete for attention. To overcome this, the authors proposed a classification model based on selective feature engineering that can identify primary objects and improve image clarity. By focusing on key objects in a scene, this model could significantly enhance the ability of patients to navigate busy or crowded environments. The ability to handle multi-object scenarios is essential for real-world applications, such as urban navigation, where the ability to identify and interact with multiple objects in the field of view can greatly improve safety and independence.

Additionally, the materials used in the construction of artificial retinas are evolving to make devices more responsive and adaptable to different environments. Guan and Chen [67] explored the use of ferroelectric polymers like P(VDF-TrFE) to improve the functionality of artificial retinas. These materials are capable of mimicking the synaptic signal transduction process, which allows for more accurate and efficient communication between the retina and the brain. Their research shows that using such materials could lead to more biocompatible and effective retinal implants. This is especially important as the prostheses need to seamlessly integrate with the body’s natural systems without causing adverse effects. The development of such materials could significantly improve the long-term efficacy of these devices, enabling them to better meet the complex demands of everyday visual tasks.

Furthermore, the integration of sensing, memory, and neuromorphic computing into a single device holds tremendous potential for creating more sophisticated retinal prosthetics. Meng et al. [68] demonstrated the development of an optoelectronic device that incorporates these capabilities to enhance artificial retinal perception. This system integrates various functions, including sensing, memory, and processing, in a way that mirrors how the human brain processes visual information. The integration of such functions into a single device is not only beneficial for enhancing the retinal prosthesis’s performance but also for enabling more scalable and efficient systems. By mimicking the brain’s ability to adapt and process visual data in real-time, these devices will be better equipped to respond to changes in the environment, such as varying lighting conditions or dynamic obstacles, offering greater flexibility and versatility for patients.

Overall, the future of ocular neuroprosthetics is increasingly intertwined with the advancements in artificial intelligence. AI’s ability to enhance task-specific visual perception, optimize image processing, and improve material efficiency holds the key to unlocking the full potential of retinal prosthetics. These innovations promise not only to restore some level of sight to individuals with incurable blindness but also to enhance their quality of life by enabling them to interact with their environments in more meaningful ways. The continued development of AI-powered solutions in this field is likely to redefine the landscape of visual neuroprosthetics, offering hope to millions of people living with severe visual impairments. Table 7 reports a brief description of the study with the focus on AI.

**Table 7:** Exploring AI Applications in Ocular Neuroprostheses: a brief description with the role of AI.

Study	Description	Focus on AI
Beyeler M, Sanchez-Garcia M (2022) [64]	The study explores the concept of a Smart Bionic Eye aimed at providing task-specific visual augmentations for individuals with incurable blindness, leveraging AI.	Focuses on deep learning-based visual augmentation to assist with real-world tasks like face recognition, navigation, and self-care, rather than replicating natural vision.
Kim S, Kwon O, et al. (2025) [65]	This research develops an information-filterable artificial retina system, integrating artificial synapses for signal perception and processing.	Utilizes AI for attention-based information processing, enabling the artificial retina to focus on significant signals while filtering out irrelevant visual information.
Mehmood A, Ko J, et al. (2024) [66]	This study addresses the challenge of object identification in artificial retina systems, proposing a classification model to improve image clarity.	AI is used to enhance image processing by selectively identifying primary objects in a multi-object environment, improving the ability to navigate complex scenarios.
Guan T, Chen S (2023) [67]	This research explores ferroelectric polymers for energy-harvesting artificial retinas, utilizing multiscale simulations to understand synaptic signal transduction.	The study focuses on improving the functionality of retinal implants by incorporating AI-driven materials, enhancing communication between the retina and brain for better visual perception.
Meng J, Wang T, et al. (2022) [68]	The research develops an integrated optoelectronic device that combines sensing, memory, and neuromorphic computing to enhance retinal prosthesis perception.	AI is incorporated to mimic the brain’s processing of visual data in real-time, enabling the prosthetic to adapt to environmental changes, such as varying lighting or obstacles.

### 4.7. Challenges in Energy Efficiency, Improving Scalability, and Ensuring Biocompatibility in Ocular Neuroprostheis

The advancement of ocular neuroprosthetics faces significant challenges that must be addressed to fully realize their potential. Among the most significant are energy efficiency, scalability, and biocompatibility. Energy efficiency is crucial for enabling these devices to operate effectively over extended periods without requiring frequent recharging or large, bulky power sources. Scalability is another key hurdle, as current devices are often limited to small-scale prototypes, and there is a need for cost-effective mass production techniques to make them widely accessible to those in need. Furthermore, ensuring biocompatibility is essential to minimize immune responses and inflammation, which could impact the long-term functionality and safety of the implants. Overcoming these challenges will be critical for advancing the field and bringing ocular neuroprosthetics to a broader range of patients. Energy efficiency remains a critical concern, as these devices often require compact power sources that support prolonged use without compromising functionality. Scalability is another pressing issue, with the need to transition from small-scale laboratory prototypes to mass-produced, cost-effective devices suitable for diverse patient populations. Additionally, ensuring biocompatibility is essential for minimizing adverse immune responses and maintaining long-term device functionality within the delicate ocular and neural environments. Addressing these challenges is pivotal for advancing the field and delivering reliable, high-performance solutions to individuals with vision impairments.

The confluence of biocompatibility and technological innovation marks a transformative era in ocular neuroprosthetics, offering unprecedented solutions for visual restoration. Key advances have emerged through materials engineered for seamless integration with biological systems, ensuring minimal immune response while achieving high functionality.

Organic mixed ionic electronic conductors (OMIECs), as explored in the work by Chen et al. [69], demonstrate remarkable potential. These materials combine ionic and electronic transport with mechanical conformability, enabling their use in artificial retinas. Their ability to mimic the ion-flux-dependent processes of natural retinas, coupled with their inherent softness, positions OMIECs as ideal candidates for interfacing with delicate ocular tissues.

Complementary to these developments, nanopatterned ceramic membranes have redefined chemical neuromodulation. Vacca et al. [52] introduced solid-state nanopores capable of spatially resolved neurotransmitter delivery. This biomimetic approach not only aligns with physiological synaptic processes but also enhances neural stimulation precision, fostering advancements in brain–machine interfaces.

Graphene-based innovations, detailed by Pang et al. [70], further underline the critical role of biocompatible materials. As a transparent and flexible conductor, graphene supports neural interfacing in artificial retinas without compromising tissue integrity. Its adaptability extends to mimicking sensory functions, such as tactile and auditory systems, heralding a broader impact on sensory prosthetics.

However, challenges persist. As Moorthy et al. [30] noted, delamination of organic photovoltaic layers in subretinal implants underscores the need for more durable solutions. Graphene-polyethylene terephthalate structures offer a promising alternative, balancing biocompatibility with enhanced stability and efficiency.

Together, these innovations not only exemplify the importance of biocompatibility in ocular neuroprosthetics but also open avenues for future research. From ionic-electronic coupling to graphene-enabled sensory restoration, the synergy between materials science and biology propels the field toward a future where vision and other senses can be effectively restored.

Table 8 reports a sketch of the overviewed studies on the biocompatibility in the field of the ocular neuroprostesis.

**Table 8:** Sketch of the studies on the biocompatibility.

Reference	Brief Description	Focus on Biocompatibility
[69]	Materials allowing ionic and electronic transport with flexibility and softness. Utilized in artificial retina designs for machine vision and human–machine interfaces.	OMIECs’ intrinsic softness ensures minimal mechanical irritation, while their ionic-electronic coupling mimics natural retinal processes.
[52]	Solid-state nanopores delivering neurotransmitters with high spatial resolution. Aimed at biomimetic chemical stimulation in brain–machine interfaces.	Chemical neuromodulation via controlled neurotransmitter diffusion offers compatibility with neural tissue, avoiding electrical overstimulation.
[70]	Flexible subretinal implants using graphene-polyethylene terephthalate (G-PET) and semiconducting single-wall carbon nanotubes (s-SWCNT).	Non-silicon, flexible designs reduce tissue stress and improve integration, while minimizing delamination risks in retinal environments.
[30]	Transparent and flexible graphene used in artificial retina, eardrums, and tactile sensors. Also applied in brain-like processors and neuromorphic computing.	Graphene’s biocompatibility and mechanical resilience enhance interaction with neural and sensory tissues, ensuring reduced immune response.

Scalability in ocular neuroprosthetics represents a cornerstone of innovation, ensuring that these technologies can move from laboratory prototypes to real-world applications capable of benefiting a broad patient population. This concept goes beyond the physical expansion of production processes—it involves the design and integration of devices that are adaptable, multifunctional, and capable of maintaining high performance across diverse environments and use cases. For neuroprosthetic systems to truly transform vision restoration and sensory augmentation, they must exhibit scalability in three critical dimensions: technological integration, functional adaptability, and production viability.

Meng et al. [68] offered a paradigm for scalable design by creating an artificial retina based on 2D Janus MoSSe. This innovative material enables the convergence of sensory, memory, and neuromorphic computing functionalities in a single device, mimicking the biological processes of the human visual system. Such integration is a hallmark of scalability, as it reduces the complexity of the system while enhancing efficiency and performance. The use of faradic electric double layers (EDLs) to modulate synaptic weight changes further exemplifies this concept by combining compact architecture with energy-efficient operations.

The scalability of ocular neuroprosthetics is not merely about technological advancements; it also addresses the practicalities of implementation. Devices must be designed to function consistently across varying environmental conditions and biological interfaces. For example, Meng et al.’s device demonstrated the ability to adapt to light variations, process visual data, and recognize handwritten digits—functions that are critical for real-world applications. These capabilities underscore the necessity of scalability to deliver consistent performance in dynamic and often unpredictable scenarios.

However, achieving scalability is not without challenges. Material stability, particularly in long-term use within biological environments, remains a significant hurdle. Additionally, the high cost associated with advanced materials and fabrication techniques can impede large-scale production and accessibility. Overcoming these barriers will require interdisciplinary collaboration, innovative material science solutions, and the development of cost-effective manufacturing processes.

Overall, scalability in ocular neuroprosthetics is both a technical and practical imperative. By ensuring that devices can adapt, integrate, and operate efficiently at scale, researchers and developers can pave the way for transformative applications in vision restoration and beyond. The work of Meng et al. [68] exemplifies the potential of scalable designs to bridge the gap between cutting-edge research and widespread clinical impact.

The management and optimization of energy dynamics in ocular neuroprosthetics have emerged as a critical aspect of advancing artificial retinal technologies. These systems aim to replicate the complex functions of the biological retina while addressing energy efficiency and sustainability to ensure long-term usability and biocompatibility.

Organic mixed ionic electronic conductors (OMIECs) offer a promising avenue for energy management in bioelectronics due to their ability to transport both ionic and electronic signals while leveraging their mechanical flexibility and biocompatibility [69]. These materials underpin the development of organic iono-optoelectronics (OIOEs), which enable the conversion of light into energy and modulate ionic-electronic coupling. Such mechanisms not only facilitate high-performance applications like artificial retinas but also provide a pathway for energy-efficient vision systems by mimicking the light-capturing processes of biological retinas.

In another approach, event-based imaging systems inspired by biological amacrine retinal circuits demonstrate energy efficiency by responding only to dynamic changes in light intensity. This is achieved using simple 1R1C circuits integrated with colloidal quantum dots, which eliminate the need for continuous data acquisition, thereby conserving energy and allowing the design of low-power artificial vision systems [58]. These circuits highlight how biomimicry can improve the energy dynamics of vision restoration technologies.

Recent advancements also explore self-driven retinomorphic systems powered by photothermoelectric effects. Ionogel-based elastomeric retinas exhibit broadband light detection and wide field-of-view capabilities without requiring external energy sources, paving the way for autonomous artificial eyes [71]. These designs integrate neuromorphic principles with energy-harvesting mechanisms, creating systems capable of dynamic optical imaging and motion tracking.

Organic heterojunction transistors are another key development in the realm of energy management. These devices emulate the energy-efficient synaptic functions of retinal neurons, enabling cost-effective and low-power systems for mobile recognition and obstacle detection [72]. By tailoring optical input properties, these systems achieve dynamic responses with minimal energy expenditure.

Multiscale simulation techniques further illuminate the energy transduction processes in artificial retinas. Ferroelectric polymers, particularly P(VDF-TrFE), facilitate the conversion of light into electrical signals through synaptic signal generation [67]. Simulations combining quantum chemistry and Monte Carlo methods provide insights into the microscopic-to-macroscopic energy dynamics, which are essential for designing efficient energy-harvesting devices.

Finally, electromagnetic modeling of dielectric resonator antenna arrays offers another dimension in energy management. These models replicate the photoreceptors of the human retina, demonstrating effective light absorption and conversion into electrochemical signals through advanced materials like graphene [73]. This approach not only enhances energy efficiency but also improves the spectral coverage and functionality of retinal implants.

Collectively, these innovations underscore the synergy between bioinspired designs and advanced material engineering in addressing the challenges of energy management in ocular neuroprosthetics. By leveraging energy-harvesting mechanisms and efficient material properties, these technologies hold the potential to revolutionize vision restoration and artificial retinal systems.

Table 9 reports a sketch of the overviewed studies on energy management in the field of ocular neuroprostesis.

**Table 9:** Sketch of the studies on the energy management in the ocular neuroprostesis.

Reference	Brief Description	Focus on Energy Management
[69]	Discusses organic mixed ionic electronic conductors (OMIECs) for bioelectronics and their application in artificial retinas.	OMIECs leverage ionic-electronic coupling for energy-efficient light modulation and vision systems. These materials facilitate light-based energy harvesting and enhance energy management in artificial retinas by mimicking biological processes.
[58]	Explores event-based imaging using simple 1R1C circuits with colloidal quantum dots to emulate retinal amacrine cell functions.	Demonstrates energy efficiency by responding only to dynamic light changes, eliminating continuous data acquisition and conserving energy in vision restoration technologies.
[71]	Introduces self-driven retinomorphic eyes using ionogel-based elastomeric retinas with photothermoelectric energy harvesting capabilities.	Achieves autonomous operation without external power sources by leveraging photothermoelectric effects. Enhances energy management through broadband light detection and neuromorphic integration for dynamic imaging and motion tracking.
[72]	Presents organic heterojunction phototransistors for artificial retinal neurons that execute synaptic functions responsive to light input.	Optimizes energy use by dynamically modulating optical inputs and executing synaptic responses efficiently. Provides a cost-effective and low-power solution for image processing and motion recognition.
[67]	Utilizes multiscale simulations to study ferroelectric polymers, particularly P(VDF-TrFE), for synaptic signal generation in artificial retinas.	Highlights energy transduction processes from light to electrical signals. Offers insights into the microscopic and macroscopic energy management required for efficient energy-harvesting devices.
[73]	Models dielectric resonator antennas as retinal photoreceptors, focusing on light absorption and electrochemical signal generation using advanced materials.	Enhances energy efficiency through graphene-based energy harvesting, enabling effective light conversion across the visible spectrum. The approach supports energy-optimized designs for retinal implants with wide spectral coverage

### 4.8. Limitations

The study has limitations due to its specific focus on an “umbrella review” of existing reviews. This approach consolidates recent, well-established themes derived from comprehensive reviews, but it does not include abstracts or conference proceedings. It aims to provide a broad overview, rather than a deep dive into individual studies or narrow topics. More targeted systematic reviews could focus on specific themes identified within the umbrella review, offering a detailed analysis of those particular aspects. This approach ensures a comprehensive understanding of the field but leaves room for further exploration through more specialized research.

## 5. Conclusions and Future Directions

In conclusion, the restoration of vision through neuroprosthetics represents a groundbreaking leap forward in medical technology, offering immense potential to improve the lives of individuals with visual impairments. The integration of artificial retinas, cortical implants, AI-enabled prosthetics, gene therapies, nanotechnology, and bioprinting has shown significant promise in enhancing both the quality and functionality of vision restoration systems. These innovations open new doors for independence, mobility, and sensory integration for those living with blindness, helping to address a range of complex visual impairments.

The promise of neuroprosthetics lies not only in the technologies themselves but also in the transformative changes they can bring to healthcare systems. AI-driven systems and the development of more efficient, personalized therapies, such as gene editing and tissue engineering, are paving the way for even more powerful prosthetic devices that can meet the unique needs of individual patients. These technologies offer a path toward higher efficiency, enhanced biocompatibility, and more sustainable, long-term integration of prosthetics. With continued interdisciplinary collaboration, there is potential to address gaps in current knowledge and improve these devices for broader, real-world use.

Importantly, emerging technologies in neuroprosthetics also bring great hope for tackling long-standing challenges. Future research is poised to explore ways to optimize the energy efficiency of these devices and improve their scalability, ensuring that they are not only effective but also accessible to a wider population. Further advancements in gene therapies, as well as in the neural dynamics of vision restoration, will likely expand the possibilities for these technologies. By fostering an inclusive, cross-disciplinary approach that balances innovation with ethical considerations, neuroprosthetics hold the promise to radically change how we approach blindness and other visual impairments.

Looking ahead, the field of neuroprosthetics offers immense promise for vision restoration, yet several challenges must be addressed to unlock its full potential. Challenges related to energy efficiency, scalability, biocompatibility, and long-term integration persist, requiring further innovation. Additionally, the complex neural dynamics involved in vision restoration and the optimization of prosthetic interfaces remain critical areas for future research.

The studies reviewed emphasize the importance of interdisciplinary collaboration in overcoming both technical and philosophical hurdles. The integration of AI and machine learning with prosthetics, along with advancements in tissue engineering and genetic therapies, offers a promising future for improving the quality of life for individuals with visual impairments. However, ongoing research is needed to refine these technologies, ensuring their long-term stability and effectiveness in real-world applications.

Regulatory, ethical, and clinical dimensions are also crucial for the successful integration of neuroprosthetic technologies into healthcare systems. Establishing adaptive regulatory frameworks, implementing informed consent protocols, ensuring robust data privacy safeguards, and promoting equitable access are essential steps. Personalizing neuroprosthetic devices to meet individual patient needs while training healthcare professionals to manage these advanced systems will be pivotal to their widespread adoption. Additionally, addressing cross-cutting challenges such as scalability, affordability, and balancing innovation with ethical considerations will require coordinated efforts from various sectors.

## Data Availability

No new data were created. Data sharing is not applicable.

## References

[1] https://www.who.int/publications/i/item/world-report-on-vision.

[2] https://www.who.int/news-room/fact-sheets/detail/assistive-technology.

[3] https://www.ncoa.org/article/how-assistive-technology-and-adaptive-equipment-help-people-living-with-vision-loss/.

[4] Wilkinson K.M., Elko L.R., Elko E., McCarty T.V., Sowers D.J., Blackstone S., Roman-Lantzy C. (2023). An Evidence-Based Approach to Augmentative and Alternative Communication Design for Individuals with Cortical Visual Impairment. Am. J. Speech-Lang. Pathol..

[5] Gunaseelaraj R., Karthikeyan S., Kumar M.N., Balamurugan T., Jagadeeshwaran A.R. (2012). Custom-made ocular prosthesis. Pharm. Bioallied Sci..

[6] Raizada K., Rani D. (2007). Ocular prosthesis. Contact Lens Anterior Eye.

[7] https://www.webmd.com/eye-health/prosthetic-eye-ocular-prosthesis.

[8] Gu J., Meng M., Cook A., Faulkner M. (2000). A study on natural movement of artificial eye implant. Robot. Auton. Syst..

[9] Wu K.Y., Mina M., Sahyoun J.Y., Kalevar A., Tran S.D. (2023). Retinal Prostheses: Engineering and Clinical Perspectives for Vision Restoration. Sensors.

[10] Ayton L.N., Barnes N., Dagnelie G., Fujikado T., Goetz G., Hornig R., Jones B.W., Muqit M.M.K., Rathbun D.L., Stingl K. (2020). An update on retinal prostheses. Clin. Neurophysiol..

[11] Liu X., Chen P., Ding X., Liu A., Li P., Sun C., Guan H. (2022). A narrative review of cortical visual prosthesis systems: The latest progress and significance of nanotechnology for the future. Ann. Transl. Med..

[12] Niketeghad S., Pouratian N. (2018). Brain Machine Interfaces for Vision Restoration: The Current State of Cortical Visual Prosthetics. Neurotherapeutics.

[13] Mirochnik R.M., Pezaris J.S. (2019). Contemporary approaches to visual prostheses. Military Med. Res..

[14] Yang J.W., Chen C.Y., Yu Z.Y., Chung J.H.Y., Liu X., Wu C.Y., Chen G.Y. (2022). An electroactive hybrid biointerface for enhancing neuronal differentiation and axonal outgrowth on bio-subretinal chip. Mater. Today Biol..

[15] Stern J.H., Tian Y., Funderburgh J., Pellegrini G., Zhang K., Goldberg J.L., Ali R.R., Young M., Xie Y., Temple S. (2018). Regenerating Eye Tissues to Preserve and Restore Vision. Cell Stem Cell.

[16] Sehic A., Guo S., Cho K.S., Corraya R.M., Chen D.F., Utheim T.P. (2016). Electrical Stimulation as a Means for Improving Vision. Am. J. Pathol..

[17] Chang K., Enayati S., Cho K.S., Utheim T.P., Chen D.F. (2021). Non-invasive electrical stimulation as a potential treatment for retinal degenerative diseases. Neural Regen. Res..

[18] https://www.sphericalinsights.com/reports/ocular-prosthesis-market.

[19] https://legacyfileshare.elsevier.com/promis_misc/ANDJ%20Narrative%20Review%20Checklist.pdf.

[20] Feldman J.A. (2024). Evolution, perception, and the mind. Cogn. Process..

[21] Wang L., Wang H., Liu J., Wang Y., Shao H., Li W., Yi M., Ling H., Xie L., Huang W. (2024). Negative Photoconductivity Transistors for Visuomorphic Computing. Adv. Mater..

[22] Kelly A.R., Glover D.J. (2024). Information Transmission through Biotic-AbioticInterfaces to Restore or Enhance Human Function. ACS Appl. Biol. Mater..

[23] Stoddart P.R., Begeng J.M., Tong W., Ibbotson M.R., Kameneva T. (2024). Nanoparticle-based optical interfaces for retinal neuromodulation: A review. Front. Cell. Neurosci..

[24] Lešták J. (2024). Visual Neuroprosthesis—Stimulation of Visual Cortical Centers in The Brain. Design of Non-Invasive Transcranial Stimulation of Functional Neurons. Czech Slovak Ophthalmol..

[25] Kurbis A.G., Kuzmenko D., Ivanyuk-Skulskiy B., Mihailidis A., Laschowski B. (2024). StairNet: Visual recognition of stairs for human-robot locomotion. Biomed. Eng. Online.

[26] Papaioannou C. (2024). Advancements in the treatment of age-related macular degeneration: A comprehensive review. Postgrad. Med. J..

[27] Kamde S.P., Anjankar A. (2023). Retinitis Pigmentosa: Pathogenesis, Diagnostic Findings, and Treatment. Cureus.

[28] Kravchenko S.V., Sakhnov S.N., Myasnikova V.V., Trofimenko A.I., Buzko V.Y. (2023). Bioprinting technologies in ophthalmology. Vestn. Oftalmol..

[29] Borchert G.A., Shamsnajafabadi H., Hu M.L., De Silva S.R., Downes S.M., MacLaren R.E., Xue K., Cehajic-Kapetanovic J. (2023). The Role of Inflammation in Age-Related Macular Degeneration-Therapeutic Landscapes in Geographic Atrophy. Cells.

[30] Pang J., Peng S., Hou C., Zhao H., Fan Y., Ye C., Zhang N., Wang T., Cao Y., Zhou W. (2023). Applications of Graphene in Five Senses, Nervous System, and Artificial Muscles. ACS Sens..

[31] Zhao D., Huang R., Gan J.M., Shen Q.D. (2022). Photoactive Nanomaterials for Wireless Neural Biomimetics, Stimulation, and Regeneration. ACS Nano.

[32] Girach A., Audo I., Birch D.G., Huckfeldt R.M., Lam B.L., Leroy B.P., Michaelides M., Russell S.R., Sallum J.M.F., Stingl K. (2022). RNA-based therapies in inherited retinal diseases. Ther. Adv. Ophthalmol..

[33] Kravchenko S.V., Sakhnov S.N., Myasnikova V.V. (2022). Modern concepts of bionic vision. Vestn. Oftalmol..

[34] Kim S., Roh H., Im M. (2022). Artificial Visual Information Produced by Retinal Prostheses. Front. Cell Neurosci..

[35] Nanegrungsunk O., Au A., Sarraf D., Sadda S.R. (2022). New frontiers of retinal therapeutic intervention: A critical analysis of novel approaches. Ann. Med..

[36] https://www.afb.org/blindness-and-low-vision/disability-rights/advocacy-resources/disability-rights-resources.

[37] Hwang J., Kim K.H., Hwang J.G., Jun S., Yu J., Lee C. (2020). Technological Opportunity Analysis: Assistive Technology for Blind and Visually Impaired People. Sustainability.

[38] https://www.who.int/standards/classifications/international-classification-of-functioning-disability-and-health#:~:text=The%20International%20Classification%20of%20Functioning,a%20list%20of%20environmental%20factors.

[39] https://www.snsinsider.com/reports/neuroprosthetics-market-2882.

[40] https://finance.yahoo.com/news/ocular-prosthesis-market-trends-challenges-105700490.html.

[41] https://news.northwestern.edu/stories/2024/12/vision-restoring-project-receives-up-to-56-million-to-fast-track-development.

[42] https://www.news-medical.net/news/20241216/Federal-funding-boosts-effort-to-make-eye-transplants-a-reality.aspx.

[43] https://www.cortigent.com/orion.

[44] https://www.ern-eye.eu/it/trials/argus-ii-retinal-prosthesis-system-post-market-study/.

[45] https://www.upmc.com/services/eye/services/retina-vitreoretinal/prima-study.

[46] https://www.visiontherapycanada.com/vision-therapy/neuro-optometric-rehabilitation-therapy/.

[47] https://www.advancedvisionsolutions.com/eye-care-services/neuro-optometric-rehabilitation/.

[48] https://www.sydney.edu.au/engineering/our-research/healthcare-engineering/implantable-neuroprosthesis.html.

[49] https://www.tomorrow.bio/it/post/la-connessione-bionica-come-le-neuroprotesi-collegano-mente-e-macchina.

[50] Nadolskis L., Turkstra L.M., Larnyo E., Beyeler M. (2024). Aligning Visual Prosthetic Development with Implantee Needs. Transl. Vis. Sci. Technol..

[51] Liang J., Li H., Chai X., Gao Q., Zhou M., Guo T., Chen Y., Di L. (2024). An audiovisual cognitive optimization strategy guided by salient object ranking for intelligent visual prosthesis systems. J. Neural Eng..

[52] Vacca F., Galluzzi F., Blanco-Formoso M., Gianiorio T., De Fazio A., Tantussi F., Stürmer S., Haq W., Zrenner E., Chaffiol A. (2024). Solid-State Nanopores for Spatially Resolved Chemical Neuromodulation. Nano Lett..

[53] Hinrichs S., Placidet L., Duret A., Authié C., Arleo A., Ghezzi D. (2024). Wide-angle simulated artificial vision enhances spatial navigation and object interaction in a naturalistic environment. J. Neural Eng..

[54] Fernandez E., Robles J.A. (2024). Advances and challenges in the development of visual prostheses. PLoS Biol..

[55] Song X., Guo T., Ma S., Zhou F., Tian J., Liu Z., Liu J., Li H., Chen Y., Chai X. (2024). Spatially Selective Retinal Ganglion Cell Activation Using Low Invasive Extraocular Temporal Interference Stimulation. Int. J. Neural Syst..

[56] Han S., Kim T., Kim C., Lee S. (2024). Design and simulation of artificial retinal stimulation IC with switched capacitor using Si nanowire optical properties. Sci. Prog..

[57] Zhu Y., Liu X., Ma J., Wang Z., Jiang H., Sun C., Jeong D.-Y., Guan H., Chu B. (2024). Wireless and Opto-Stimulated Flexible Implants: Artificial Retina Constructed by Ferroelectric BiFeO_3_-BaTiO_3_/P(VDF-TrFE) Composites. ACS Appl. Mater. Interfaces.

[58] Al Mahfuz M.M., Islam R., Ko D.K. (2024). Artificial Amacrine Retinal Circuits. ACS Appl. Mater. Interfaces.

[59] Jensen N., Charles Chen Z., Kochnev Goldstein A., Palanker D. (2024). Accelerated Simulation of Multi-Electrode Arrays Using Sparse and Low-Rank Matrix Techniques. bioRxiv.

[60] van Velthoven E.A.M., van Stuijvenberg O.C., Haselager D.R.E., Broekman M., Chen X., Roelfsema P., Bredenoord A.L., Jongsma K.R. (2022). Ethical implications of visual neuropros-theses—A systematic review. J. Neural Eng..

[61] Stronks H.C., Dagnelie G. (2014). The functional performance of the Argus II retinal prosthesis. Expert Rev. Med. Devices.

[62] Ramirez K.A., Drew-Bear L.E., Vega-Garces M., Betancourt-Belandria H., Arevalo J.F. (2023). An update on visual prosthesis. Int. J. Retin. Vitr..

[63] https://www.centropiaggio.unipi.it/sites/default/files/course/material/papers_visual_prostheses.pdf.

[64] Beyeler M., Sanchez-Garcia M. (2022). Towards a Smart Bionic Eye: AI-powered ar-tificial vision for the treatment of incurable blindness. J. Neural Eng..

[65] Kim S., Kwon O., Kim S., Jang S., Yu S., Lee C.H., Choi Y.Y., Cho S.Y., Kim K.C., Yu C. (2025). Modulating synaptic plasticity with metal-organic framework for information-filterable artificial retina. Nat. Commun..

[66] Mehmood A., Ko J., Kim H., Kim J. (2024). Optimizing Image Enhancement: Feature Engineering for Improved Classification in AI-Assisted Artificial Retinas. Sensors.

[67] Guan T., Chen S. (2023). Multiscale Simulations on Synaptic Signal Transduction of Energy-Harvesting P(VDF-TrFE)-Based Artificial Retina. J. Phys. Chem. B.

[68] Meng J., Wang T., Zhu H., Ji L., Bao W., Zhou P., Chen L., Sun Q.Q., Zhang D.W. (2022). Integrated In-Sensor Computing Optoelectronic Device for Environment-Adaptable Artificial Retina Perception Application. Nano Lett..

[69] Chen K., Song I., You L., Mei J. (2025). Organic Iono-Optoelectronics: From Electrochromics to Artificial Retina. Acc. Chem. Res..

[70] Moorthy V.M., Rathnasami J.D., Srivastava V.M. (2023). Design Optimization and Characterization with Fabrication of Nanomaterials-Based Photo Diode Cell for Subretinal Implant Application. Nanomaterials.

[71] Luo X., Chen C., He Z., Wang M., Pan K., Dong X., Li Z., Liu B., Zhang Z., Wu Y. (2024). A bionic self-driven retinomorphic eye with ionogel photosynaptic retina. Nat. Commun..

[72] Wang S., Shi X., Gong J., Liu W., Jin C., Sun J., Peng Y., Yang J. (2024). Artificial Retina Based on Organic Heterojunction Transistors for Mobile Recognition. Nano Lett..

[73] NoroozOliaei M., Riazi Esfahani H., Abrishamian M.S. (2023). Modeling of dielectric resonator antenna array for retina photoreceptors. Heliyon.

